# 2,3,5,4′-tetrahydroxystilbene-2-O-β-D-glucoside ameliorates bleomycin-induced pulmonary fibrosis *via* regulating pro-fibrotic signaling pathways

**DOI:** 10.3389/fphar.2022.997100

**Published:** 2022-10-04

**Authors:** Tsung-Teng Huang, Chuan-Mu Chen, Lih-Geeng Chen, Ying-Wei Lan, Tse-Hung Huang, Kong Bung Choo, Kowit-Yu Chong

**Affiliations:** ^1^ Department of Medical Biotechnology and Laboratory Science, College of Medicine, Chang Gung University, Taoyuan, Taiwan; ^2^ Graduate Institute of Biomedical Sciences, Division of Biotechnology, College of Medicine, Chang Gung University, Taoyuan, Taiwan; ^3^ Department of Life Sciences, Agricultural Biotechnology Center, National Chung Hsing University, Taichung, Taiwan; ^4^ The iEGG and Animal Biotechnology Center and the Rong Hsing Research Center for Translational Medicine, National Chung Hsing University, Taichung, Taiwan; ^5^ Department of Microbiology, Immunology and Biopharmaceuticals, National Chiayi University, Chiayi, Taiwan; ^6^ Division of Pulmonary Biology, The Perinatal Institute of Cincinnati Children’s Research Foundation, Cincinnati, OH, United States; ^7^ Department of Traditional Chinese Medicine, Chang Gung Memorial Hospital at Keelung, Keelung, Taiwan; ^8^ Centre for Stem Cell Research, Faculty of Medicine and Health Sciences, Universiti Tunku Abdul Rahman, Kajang, Selangor, Malaysia; ^9^ Hyperbaric Oxygen Medical Research Lab, Bone and Joint Research Center, Chang Gung Memorial Hospital at Linkou, Taoyuan, Taiwan

**Keywords:** THSG, bleomycin, pulmonary fibrosis, TGF-β1, autophagy

## Abstract

2,3,5,4′-Tetrahydroxystilbene-2-O-β-D-Glucoside (THSG) is the main active ingredient extracted from *Polygonum multiflorum* Thunb. (PMT), which has been reported to possess extensive pharmacological properties. Nevertheless, the exact role of THSG in pulmonary fibrosis has not been demonstrated yet. The main purpose of this study was to investigate the protective effect of THSG against bleomycin (BLM)-induced lung fibrosis in a murine model, and explore the underlying mechanisms of THSG in transforming growth factor-beta 1 (TGF-β1)-induced fibrogenesis using MRC-5 human lung fibroblast cells. We found that THSG significantly attenuated lung injury by reducing fibrosis and extracellular matrix deposition. THSG treatment significantly downregulated the expression levels of TGF-β1, fibronectin, α-SMA, CTGF, and TGFBR2, however, upregulated the expression levels of antioxidants (SOD-1 and catalase) and LC3B in the lungs of BLM-treated mice. THSG treatment decreased the expression levels of fibronectin, α-SMA, and CTGF in TGF-β1-stimulated MRC-5 cells. Conversely, THSG increased the expression levels of SOD-1 and catalase. Furthermore, treatment of THSG profoundly reduced the TGF-β1-induced generation of reactive oxygen species (ROS). In addition, THSG restored TGF-β1-induced impaired autophagy, accompany by increasing the protein levels of LC3B-II and Beclin 1. Mechanism study indicated that THSG significantly reduced TGF-β1-induced increase of TGFBR2 expression and phosphorylation of Smad2/3, Akt, mTOR, and ERK1/2 in MRC-5 cells. These findings suggest that THSG may be considered as an anti-fibrotic drug for the treatment of pulmonary fibrosis.

## Introduction

Idiopathic pulmonary fibrosis (IPF) is characterized by progressive and lethal symptoms of interstitial lung disease with an unclear etiology. Mortality rate of IPF is higher than many cancers, with a median survival of 3–4 years following diagnosis (Sauleda et al., 2018). Atrophic alveolar epithelial damage is associated with proliferation and activation of fibroblasts and myofibroblasts, resulting in aberrant deposition of extracellular matrix (ECM) components and extensive remodeling of lung architecture (Richeldi et al., 2017). Conventional pharmacological therapies for pulmonary fibrosis include corticosteroids alone or in combination with other immunosuppressive and immunomodulatory agents; however, the curative effects of these drugs is limited and can cause a number of side effects, such as nausea, anorexia, dyspepsia, and skin rash (Bouros and Antoniou, 2005; Richeldi et al., 2017; Gibson et al., 2020). So far, the U.S. Food and Drug Administration has approved pirfenidone and nintedanib for the use of mild-to-moderate IPF therapy, but these two drugs have only been able to retard the progression of pulmonary fibrosis (Raghu et al., 2015). Therefore, exploration of novel effective therapeutic agents for IPF is still needed.

A previous study has shown that there are many cytokines and growth factors involving in the development of fibrotic disorders (Allen and Spiteri, 2002). Among them, transforming growth factor-beta 1 (TGF-β1) is the most studied profibrotic cytokine that is involved in the development of pulmonary fibrosis (Sime et al., 1997). This cytokine promotes fibroblasts proliferating and differentiating into active myofibroblasts, which leads to excessive collagen synthesis and ECM protein accumulation, and contribute to the recruitment of inflammatory cells (Fernandez and Eickelberg, 2012). Differentiation of fibroblasts into myofibroblasts is identified by the expression of alpha-smooth muscle actin (α-SMA), and both fibroblasts and myofibroblasts are primary sources of ECM proteins such as fibronectin and collagen (Jarman et al., 2014). Moreover, an increased expression level of TGF-β1 in the lungs was found in lung fibrosis animal models and patients with IPF (Tanaka et al., 2010; Bao et al., 2014). Transient overexpression of TGF-β1 by using adenoviral vectors carrying an active TGF-β1 construct induced prolonged severe interstitial and pleural fibrosis in rat lungs (Sime et al., 1997). In addition, epithelium-specific deletion of TGF-β receptor type II protects mice from bleomycin (BLM)-induced pulmonary fibrosis, further implying that the TGF-β signaling pathway plays a central role for alveolar epithelium in fibrogenesis (Li et al., 2011). Therefore, inhibiting of the fibrogenic cytokine TGF-β1 production and blocking the ECM process represent a potential approach for pulmonary fibrosis therapy.

TGF-β exerts its function through binding to heteromeric receptor complexes composed of type I and type II receptors, and then triggers intracellular signaling cascades including Smad-dependent and Smad-independent pathways (Koli et al., 2008; Ahn et al., 2011). In the Smad-dependent pathway, TGF-β binds first to the type II receptor (TGFBR2), after which the type I receptor (TGFBR1) is recruited to the complex and activated by a phosphorylation event. Subsequently, the activated TGFBR1 promotes the phosphorylation of Smad2 and Smad3 that form complexes with Smad4 and allow the complex to translocate from the cytosol to the nucleus and regulate the transcription of downstream genes (Shi and Massagué, 2003). Although the Smad pathway is critical for TGF-β-mediated signaling, several studies show that TGF-β activates non-Smad signal pathways, including mitogen-activated protein kinases (MAPKs), Rho-like GTPase, and phosphatidylinositol 3-kinase (PI3K)/Akt pathways. Three well-established signaling molecules of MAPK pathways regarding TGF-β-mediated signaling consist of extracellular signal-regulated kinase 1/2 (ERK1/2), stress-activated protein kinase or c-Jun N-terminal kinase (SAPK or JNK), and p38 MAPK (Wilkes et al., 2005; Zhang, 2009; Luo et al., 2014).

Autophagy is a critical cellular homeostatic process, which plays an essential role in removing denatured proteins as well as damaged cellular components. Many cell hyperproliferative disorders, including pulmonary fibrosis, are considered to be alleviated by autophagy activation (Haspel and Choi, 2011; Cabrera et al., 2015). Autophagy defects promote the expression of α-SMA and ECM proteins (Patel et al., 2012). Studies have shown that microtubule-associated protein 1 light chain 3 beta (LC3B) expression is downregulated in lung fibrosis tissues of patients, indicating autophagy activity is decreased (Wang et al., 2018). The activation of autophagy in mice after BLM injury can reduce collagen deposition in the lungs and increase survival rate (Araya et al., 2013). Furthermore, treatment of TGF-β1 in human lung fibroblasts inhibits autophagic processes, which may promote fibrogenesis in IPF (Patel et al., 2012). Silencing of LC3 and Beclin 1 genes or pharmacological inhibition of autophagy in human lung fibroblasts augments TGF-β1-induced expression of fibronectin and α-SMA (Patel et al., 2012). These results indicate that TGF-β1 is also involved in the pathogenesis of IPF via inhibiting autophagy processes and regulating fibrotic processes. In addition, the intracellular pathway PI3K/Akt suppresses autophagy through its downstream mammalian target of rapamycin (mTOR) (Jung et al., 2010). A recent study has found that activation of the mTOR pathway in lung epithelial cells promotes epithelial–mesenchymal transition (EMT) and lung injury, leading to acceleration of pulmonary fibrosis (Saito et al., 2020). Lung fibrosis is alleviated by inhibition of the PI3K/Akt/mTOR signaling pathway in the BLM-induced pulmonary fibrosis animal model (Chitra et al., 2015). Therefore, interventions aimed at restraining the activation of Smad, MAPK, and PI3K/Akt/mTOR signaling pathways provides an attractive strategy for a potent agent against pulmonary fibrosis.

2,3,5,4′-tetrahydroxystilbene-2-O-β-D-glucoside (THSG) is one of the major bioactive constituents extracted from *Polygonum multiflorum* Thunb. (PMT; Ho Shou Wu in Chinese). Previous studies have validated that THSG exhibits various pharmacologic effects, including cardiovascular protection, hepatoprotection, neuroprotection, the promotion of hair growth, memory enhancement, anti-cancer, anti-aging, anti-osteoporosis, anti-oxidation, anti-inflammation and so on (Büchter et al., 2015; Ling and Xu, 2016; Yu et al., 2017; Wu et al., 2020; Wang et al., 2022). THSG has also been shown to exhibit anti-fibrotic effect on pressure overloaded-induced cardiac fibrosis (Peng et al., 2016). However, the protective role of THSG in pulmonary fibrosis and its potential mechanisms has not been elucidated.

The aim of the current study is to decipher the *in vitro* and *in vivo* protective efficacy of THSG against pulmonary fibrosis with molecular mechanism elucidation. Thereafter, the underlying mechanisms of THSG against TGF-β1-induced fibrotic process of human lung fibroblasts and alveolar epithelial cells were further examined. These results provide compelling evidence for the therapeutic potential of THSG in pulmonary fibrosis.

## Materials and methods

### Reagents and antibodies

The recombinant human TGF-β1 was obtained from R&D Systems (Minneapolis, MN, United States). Bovine serum albumin (BSA), Bleomycin (BLM) sulfate from *Streptomyces verticillus*, hydroxychloroquine (HCQ) sulfate, and 2,3,5,4′-tetrahydroxystilbene-2-O-β-D-glucoside (THSG) were purchased from Sigma-Aldrich (Saint Louis, MO, United States). THSG was dissolved in ddH_2_O at 5 mg/ml concentration as a stock solution that was stored at –20°C. Minimum essential medium (MEM), Dulbecco’s modified Eagle’s medium (DMEM), sodium pyruvate (100 mM), fetal bovine serum (FBS), and antibiotic-antimycotic (100×) were purchased from Life Technologies (Grand Island, NY, United States). Primary antibodies against Akt, phospho (p)-Akt (Ser473), SAPK/JNK, p-SAPK/JNK (Tyr183/Tyr185), ERK1/2, p-ERK1/2 (Thr202/Tyr204), p38 MAPK, p-p38 MAPK (Thr180/Tyr182), mTOR, p-mTOR (Ser2448), Smad2/3, and p-Smad2 (Ser465/467)/Smad3 (Ser423/425) were purchased from Cell Signaling Technology (Beverly, MA, United States). Antibodies specific to Beclin 1, catalase, connective tissue growth factor (CTGF), E-cadherin, LC3B, and TGFBR2 were purchased from Proteintech (Chicago, IL, United States). Fibronectin, SOD-1, and β-actin antibodies were supplied by Santa Cruz Biotechnology (Dallas, TX, United States). TGFBR1 antibody was obtained from Affinity Biosciences (Cincinnati, OH, United States). N-cadherin and α-SMA antibodies were purchased from ABclonal Biotechnology (Wuhan, Hubei, China). Horseradish peroxidase (HRP)-conjugated goat anti-rabbit/mouse IgG secondary antibodies were supplied by Santa Cruz Biotechnology.

### Murine model of bleomycin-induced pulmonary fibrosis

Male C57BL/6 mice (8 weeks of age; body weight, 22–25 g) were obtained from the National Laboratory Animal Center (Taipei, Taiwan). The animals were housed in an air-conditioned animal facility with constant temperature and humidity, a 12/12 h light/dark cycle, and fed with a commercial diet and tap water *ad libitum*. All experimental procedures were according to the guidelines of Institutional Animal Care and Use Committee approved by Chang Gung University. The mice were randomly allocated into four groups (*n* = 6 per group) as follows: 1) control group, 2) BLM group, 3) BLM + THSG (10 mg/kg) group, and 4) BLM + THSG (30 mg/kg) group. The BLM sulfate stock was dissolved in sterile phosphate-buffered saline (PBS) at 10 mg/ml and stored in small aliquots at 4°C. Mice were anesthetized with isoflurane (Abbott Laboratories, Abbott Park, IL, United States) and BLM was administered intratracheally (1.5 mg/kg) in PBS on day 0 as previously described (Huang et al., 2015) while control animals receive an equal volume of sterile PBS. The mice of BLM + THSG groups were received THSG dissolved in 0.1 ml of sterile PBS by oral gavage five times a week, starting from the third to the 21st day after BLM instillation. On day 21, all mice were sacrificed and lung tissues were collected for the following experiments.

### Culture of lung fibroblasts and alveolar epithelial cells

Human lung fibroblast cell line MRC-5 was obtained from the Bioresource Collection and Research Center (BCRC, Hsinchu, Taiwan). Human lung alveolar epithelial cell line A549 was obtained from the American Type Culture Collection (Manassas, VA, United States). MRC-5 and A549 cells were cultured in MEM and DMEM respectively, supplemented with 10% FBS, 1% sodium pyruvate, and 1% antibiotic-antimycotic solution. Cells were incubated in a humidified chamber at 37°C with 95% air and 5% CO_2_ environment. In this study, the MRC-5 cells were pretreated with various concentrations of THSG (50 and 100 μg/ml) for 24 h. Afterward, exogenous 2.5 ng/ml of TGF-β1 was administered for 1, 6, and 24 h. Additionally, the A549 cells were pretreated with 50 and 100 μg/ml of THSG for 2 h, and then stimulated with 5 ng/ml of TGF-β1 for 30 min, 1, 3, and 48 h.

### MTT assay

Effect of THSG on cell viability of MRC-5 cells was assessed using the *In Vitro* Toxicology Assay Kit, MTT based from Sigma-Aldrich as described previously (Huang et al., 2014). Briefly, a density of 2 × 10^4^ cells per well was cultured in 96-well plates. After 24 h, cells were treated with various concentrations (50, 100, and 200 μg/ml) of THSG for either 24 or 48 h. According to the manufacturer’s instructions, the cells were incubated with MTT solution at a concentration of 0.5 mg/ml for 4 h. Finally, formazan crystals were solubilized with the MTT solubilization solution. The absorbance of the mixture was measured at 570 nm with a SpectraMax M5 multi-mode microplate reader (Molecular Devices, Sunnyvale, CA, United States).

### Measurement of reactive oxygen species production

MRC-5 cells were seeded in 96-well black wall/clear bottom plates at a density of 2×10^4^ cells per well for overnight adhesion. Before experiments, cells were serum starved for 24 h, followed by treatment with THSG (50 and 100 μg/ml) for further incubation for 24 h. The cells were subsequently incubated with 2.5 ng/ml of TGF-β1 for an additional 24 h. ROS production in cells was measured using the Total ROS/Superoxide detection kit (Enzo Life Sciences, Farmingdale, NY, United States) following the manufacturer’s instructions.

### Autophagy analysis

Autophagy was determined using an Autophagy Assay Kit (Sigma-Aldrich) following the manufacturer’s instructions. In brief, MRC-5 cells were passaged and treated as mentioned in the ROS production assay. After the treatment, cells were incubated with an autophagosome detection reagent working solution (diluting 20 μl of the 500× autophagosome detection reagent with 10 ml of the stain buffer) for 45 min at 37°C. Next, cells were washed with the wash buffer for four times. The fluorescence intensity was measured at a wavelength of 520 nm using excitation wavelength at 360 nm. Relative fluorescence intensities were used to measure Intracellular autophagosome production.

### Histopathological examination

The lung tissues of mice were fixed with 4% paraformaldehyde overnight and embedded in paraffin wax. The paraffin-embedded specimens were then sectioned at 5 μm and stained with hematoxylin and eosin (H&E, Sigma-Aldrich) for evaluating fibrotic lesions. The Ashcroft score was used for semi-quantitative assessment of lung fibrotic changes (Ashcroft et al., 1988). For visualization of collagen deposition, Masson’s trichrome staining (Trichrome Stain (Masson) Kit, Sigma-Aldrich) was performed to examine the density and magnitude of collagen fibers in the lungs, an index of lung fibrosis.

### Estimation of collagen content by sircol collagen assay

The levels of soluble collagen in the lung tissues were determined by the Sircol Collagen Assay Kit according to the manufacturer’s instructions (Biocolor Ltd., Carrickfergus, United Kingdom). In brief, the lung tissue samples were incubated with acid neutralizing reagent and isolation and concentration reagent and then centrifuged. Lung extracts were incubated with Sircol dye reagent and centrifuged to precipitate the collagen-dye complex pellets. The Sircol dye was released from the pellets by using an alkali reagent (1 N NaOH, Sigma-Aldrich) and the absorbance measured at 550 nm using a SpectraMax M5 multi-mode microplate reader (Molecular Devices).

### Immunohistochemical staining analysis

The lung tissue sections (5 μm thick) were deparaffinized in xylene and were treated with graded ethanol solutions for rehydration. To retrieve antigens, the sections were microwave-heated in citrate buffer (10 mM of sodium citrate, pH 6.0) and kept to boil for 10–15 min. The endogenous peroxidase were quenched by 3% hydrogen peroxide (H_2_O_2_) for 15 min at room temperature. After blocked with goat serum for 30 min, the sections were further incubated with primary antibodies specific to fibronectin, α-SMA, CTGF, TGF-β1, TGFBR1, TGFBR2, SOD-1, catalase, and LC3B overnight at 4°C, prior to incubation in EnVision Detection Systems (DAKO, Glostrup, Denmark) following the manufacturer’s instructions. Subsequently, the bound antibodies were visualized with 3,3**′**-diaminobenzidine (DAB), counterstained with hematoxylin, dehydrated by increasing the concentration of ethanol concentration gradually, cleared with xylene, and mounted in glycerol-gelatin. Images from stained slides were obtained using HistoFAXS (Tissue FAX Plus; Tissue Gnostics, Vienna, Austria).

### RNA Extraction and Quantitative Real-Time Polymerase Chain Reaction

The total RNA from lung tissues were extracted using RNeasy Mini Kit (Qiagen, Valencia, CA, United States) following the protocol provided by the manufacturer. The reverse transcription of 1 μg of total RNA was performed in a solution (final volume of 20 μL) containing oligo dT primer, dNTP, and reverse transcriptase (SuperScript III, Invitrogen, Carlsbad, CA, United States). The sequences of the mouse gene-specific primers are shown as follows: forward primer 5′-TAT​GGG​GAC​AAT​ACA​CAA​GGC​T-3′, reverse primer CGG​GCC​ACC​ATG​TTT​CTT​AGA-3**′** for SOD-1; forward primer 5**′**-AGC​GAC​CAGATG​AAG​CAG​TG-3**′,** reverse primer 5**′**-TCC​GCT​CTC​TGT​CAA​AGT​GTG-3**′** for catalase; forward primer 5′-TTG​CTT​CAG​CTC​CAC​AGA​GA-3′, reverse primer 5′-TGG​TTG​TAG​AGG​GCA​AGG​AC-3′ for TGF-β1; forward primer 5′-GCG​AGA​AGATGA​CCC​AGA​TC-3′, reverse primer 5′-CCA​GTG​GTA​CGG​CCA​GAG​G-3′ for β-actin. Quantitative RT-PCR was conducted with Roche SYBR Green Master Mix (Mannheim, Germany) according to our previously described protocol (Huang et al., 2013). Relative quantification of gene expression was calculated using a manufacturer-provided mathematical model. β-actin was used as an internal standard to normalize the expression level of each mRNA. Fold expression is based on at least three to five biological replicates for each treatment group.

### Western blot analysis

After treatment with THSG and TGF-β1, the cells were harvested at the indicated times and lysed in radioimmunoprecipitation assay (RIPA) lysis buffer (Millipore Corporation, Billerica, MA, United States) containing a cocktail of protease and phosphatase inhibitor (Thermo Fisher Scientific, Waltham, MA, United States). Protein extraction and Western blot analysis were performed as described previously (Huang et al., 2014). Equal amounts of total protein from each sample were separated by 8%–12% sodium dodecyl sulfate-polyacrylamide gel electrophoresis (SDS-PAGE) and further transferred to polyvinylidene difluoride (PVDF) membranes (Millipore). Membranes were blocked with 5% non-fat skim milk or BSA in a TBST solution containing Tris-buffered saline with 0.1% Tween-20 for 1 h at room temperature, and then probed with the indicated primary antibodies overnight at 4°C. After washing steps, the membranes were probed with HRP-conjugated specific secondary antibodies for 90 min at room temperature. Finally, the immunoreactive bands were detected using the enhanced chemiluminescence (ECL) detection system (Millipore). The images of bands were quantified using the software ImageJ (National Institutes of Health, Bethesda, MD, United States) for densitometric analysis.

### Statistical analysis

The data are presented as means ± standard error of the mean (SEM) from at least three different experiments. The two-tailed Student’s *t*-test was used for comparing the differences between two groups, and one-way analysis of variance (ANOVA) followed by Dunnett’s post hoc test was used for multiple comparisons. A value of *p <* 0.05 was considered statistically significant.

## Results

### 2,3,5,4′-tetrahydroxystilbene-2-O-β-D-glucoside attenuates bleomycin-induced pulmonary fibrosis in mice

**FIGURE 1:**
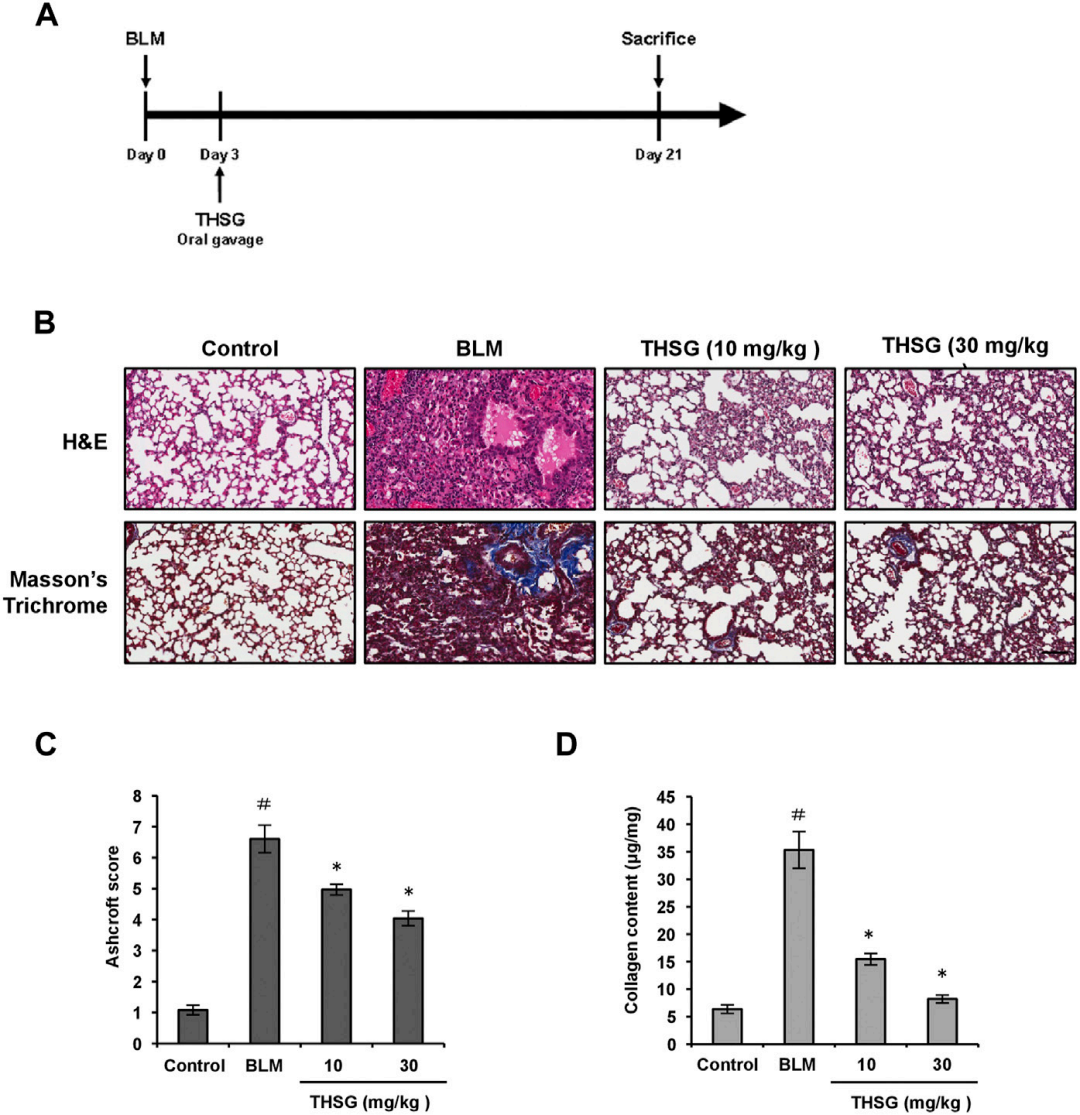
Effect of THSG on BLM-induced pulmonary fibrosis in mice. **(A)** Experimental design: Mice were intratracheally injected with BLM (1.5 mg/kg in 50 μL of PBS) at day 0. The treatment groups received orally with THSG (10 or 30 mg/kg) once a day, five times a week from day 3 to day 20. Mice were sacrificed on day 21 and lung samples were collected for further analysis. **(B)** Representative photographs of H&E and Masson’s trichrome staining of lung tissue sections in the indicated groups. Scale bar: 100 μm. **(C)** Ashcroft fibrosis scores were used to evaluate the degree of lung fibrosis. **(D)** Detection of collagen content in the lung tissues of mice from different experimental groups by the Sircol collagen assay. Data are represented as the mean ± SEM (*n* = 6 in each group). ^#^ *p* < 0.05 compared with vehicle-treated control group. *p < 0.05 compared with BLM group.

The mouse model of BLM-induced pulmonary fibrosis was established, and mice were treated with THSG after intratracheal installation of BLM (Figure 1A). To analyze the therapeutic effect of THSG on BLM-induced pulmonary fibrosis in mice, H&E staining, Masson’s trichrome staining, and Sircol collagen assay were performed in the lung tissue sections of mice from each group. H&E staining results revealed that severely thickened alveolar walls, disturbed lung architecture, and evident fibrous hyperplasia in the lung interstitium was induced by the BLM instillation, and these pathological changes were markedly improved by the oral administration of THSG (Figure 1B). Masson’s Trichrome staining showed the accumulation of ECM i.e. Collagen among the BLM-induced fibrosis model, which was significantly reduced when treated with THSG in a dose-dependent manner (Figure 1B). Consequently, Ashcroft score was used to quantify the overall grade of the fibrotic changes in the lungs (Figure 1C). The scores of the mice administered with BLM were significantly elevated compared to the control group. THSG (10 and 30 mg/kg) treatment strikingly reduced the Ashcroft score compared to the BLM model group. Similarly, analysis of collagen content in the lung tissues by Sircol collagen assay confirmed that the BLM-induced elevation of collagenous protein was clearly suppressed by THSG in a dose-dependent manner, as shown in Figure 1D.

### 2,3,5,4′-tetrahydroxystilbene-2-O-β-D-glucoside inhibits transforming growth factor-beta 1-induced expression of fibrotic markers in MRC-5 human lung fibroblast cells

**FIGURE 2:**
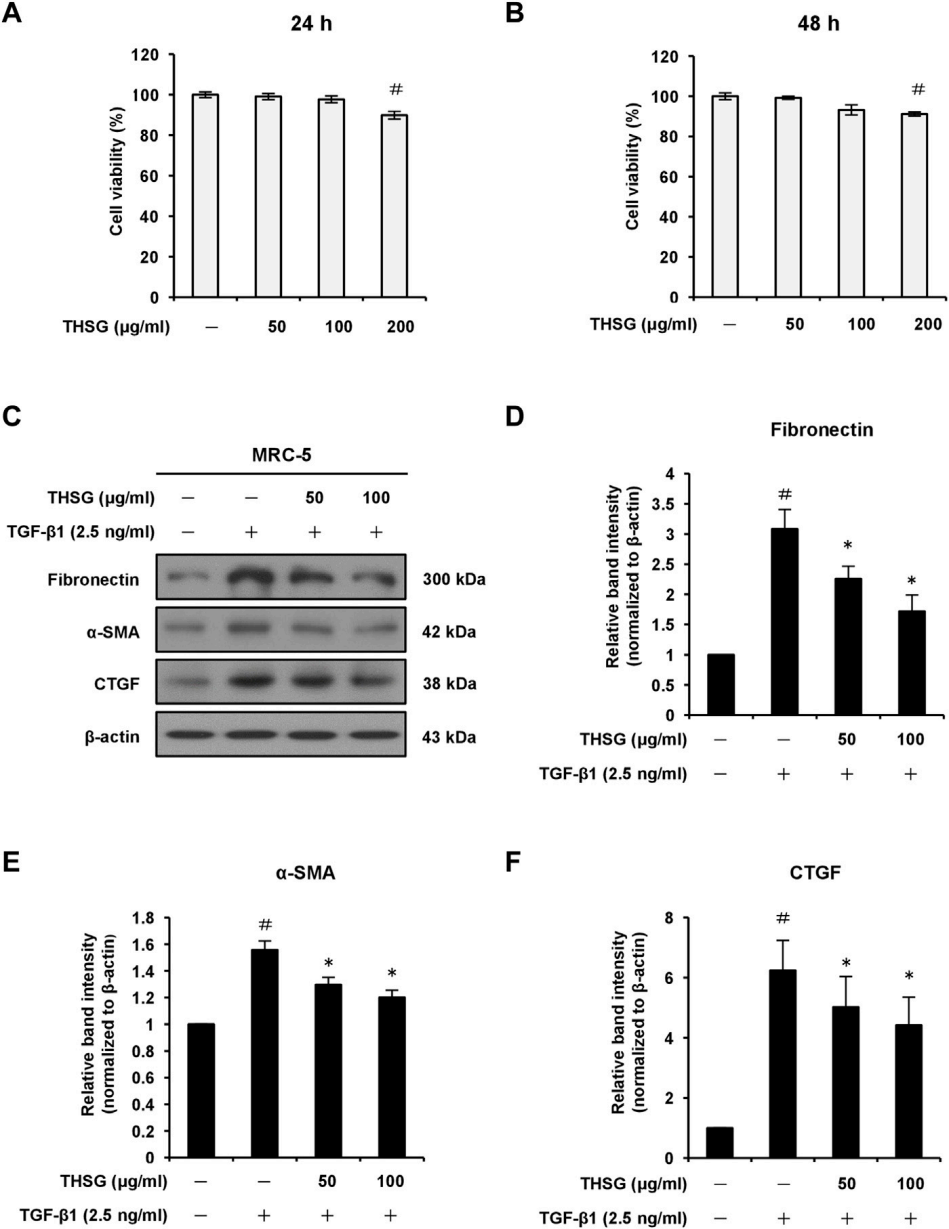
Effect of THSG on protein expression levels of fibronectin, α-SMA, and CTGF in TGF-β1-stimulated MRC-5 human lung fibroblast cells. The cells were treated with different concentrations (50, 100, and 200 μg/ml) of THSG for **(A)** 24 h or **(B)** 48 h and viability was measured by the MTT assay. **(C)** Following 24 h of serum starvation, cells were treated with varying concentrations (50 and 100 μg/ml) of THSG for 24 h, and then incubated with 2.5 ng/ml of TGF-β1 for an additional 24 h. The protein expression levels of fibrotic markers in whole cell lysates were determined by Western blotting and the β-actin was used as a loading control. The relative protein levels of **(D)** fibronectin, **(E)** α-SMA, and **(F)** CTGF were quantified by densitometry and normalized to β-actin. The results were expressed as relative units. Data are represented as the mean ± SEM of three independent experiments. ^#^ *p* < 0.05 compared with untreated group. *p < 0.05 compared with TGF-β1-treated group.

To test the impact of THSG on cell viability, MRC-5 cells were treated with 50, 100, and 200 μg/ml THSG for 24 and 48 h. The results of MTT assay showed an obvious reduction in cell viability after 24 or 48 h incubation with 200 μg/ml THSG compared with untreated control cells; however, cell viability of MRC-5 cells did not significant decrease at the concnntration of 50 or 100 μg/ml THSG (Figures 2A,B). Hence, 50 and 100 μg/ml of THSG treatments were chosen for the following experiments. To further determine the role of THSG in pulmonary fibrosis, we examined the effect of THSG in TGF-β1-induced myofibroblast differentiation and ECM deposition *in vitro*. As shwon in Figures 2C,D, Stimulation with 2.5 ng/ml of TGF-β1 significantly upregulated the protein level of fibronectin, while pretreatment of THSG (50 and 100 μg/ml) for 24 h dose-dependently downregulated TGF-β1-induced the expression of fibronection in MRC-5 cells. We also observed that adddition of TGF-β1 significantly increased the protein expression of the myofibroblast activation marker α-SMA, and THSG inhibited TGF-β1-induced the expression of α-SMA in MRC-5 cells (Figures 2C,E). Additionally, CTGF is a downstream mediator of TGF-β1 and has been suggested to play a key role in TGF-β-induced connective tissue cell proliferation and ECM deposition, leading to promotion and maintainace of fibrogenesis (Kothapalli et al., 1997). We assessed the protein level of CTGF by THSG after stimulation of TGF-β1. Western blot analysis and quantative results demonstrated that the expression of CTGF was dramatically elevated by treatment of TGF-β1, and pretreatment of THSG for 24 h significantly attenuated TGF-β1-induced CTGF expession in MRC-5 cells (Figures 2C,F). In short, THSG can inhibit the proliferation and activation of fibroblasts.

### 2,3,5,4′-tetrahydroxystilbene-2-O-β-D-glucoside suppresses transforming growth factor-beta 1-induced expression of epithelial–mesenchymal transition related markers in A549 human lung alveolar epithelial cells

TGF-β induces injured alveolar epithelial cells to undergo EMT, which contributes to the expansion of myofibroblasts causing the progression of fibrosis (Xu et al., 2009). Therefore, we examined whether THSG could influence TGF-β1-induced EMT related protein expression in A549 cells. As shown in Supplementary Figures S1A,B, Western blot analysis and quantative results showed that the protein level of the epithelial phenotype marker E-cadherin was significantly decreased after stimulation of 5 ng/ml TGF-β1 for 48 h, while pretreatment with 100 μg/ml of THSG for 2 h profoundly increased the expression of E-cadherin in A549 cells. In contrast, TGF-β1 treatment upregulated the protein levels of the mesenchymal phenotype markers N-cadherin and fibronectin compared with the untreated cells. In cells pretreated with THSG, the expression levels of N-cadherin and fibronectin proteins were obviously repressed compared to cells in the TGF-β1-treated group (Supplementary Figures S1A,C,D).

### 2,3,5,4′-tetrahydroxystilbene-2-O-β-D-glucoside suppresses TGFBR2 expression and transforming growth factor-beta 1-induced Smad2/3 signaling pathway

**FIGURE 3:**
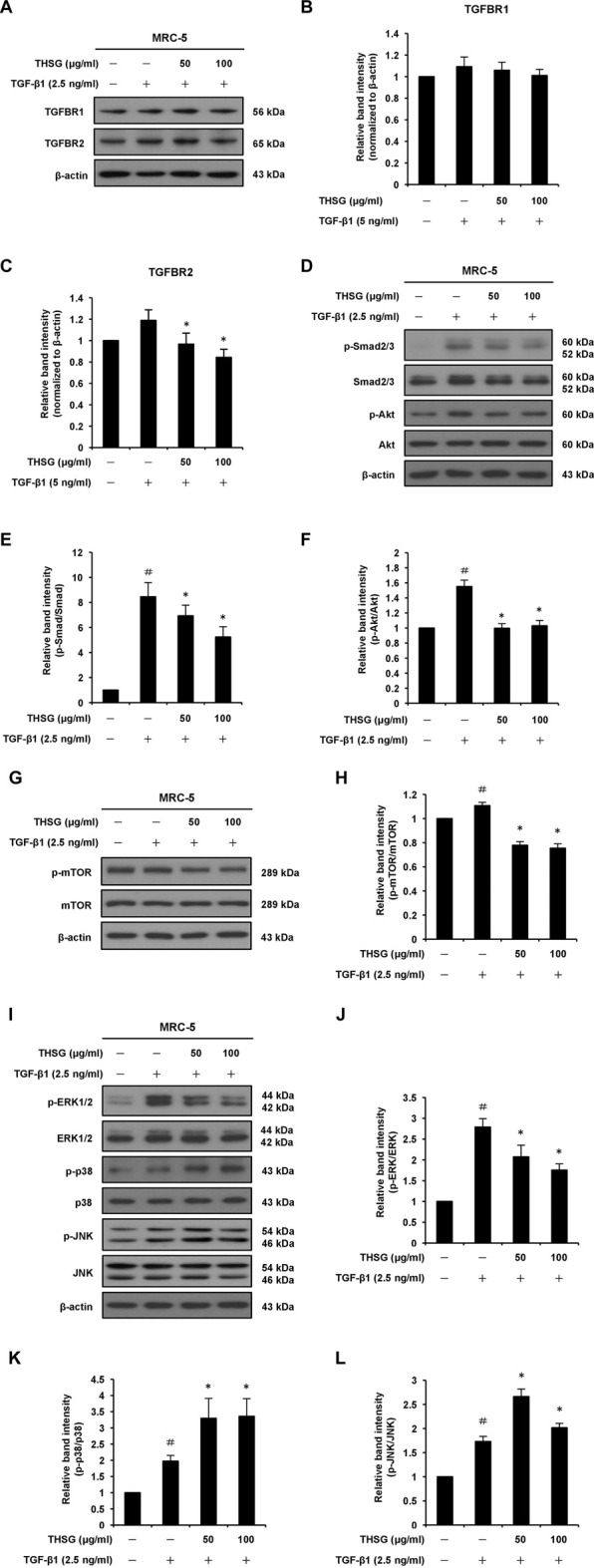
Effect of THSG on TGF-β receptors and TGF-β1-induced Smad-dependent and -independent signaling pathways in MRC-5 human lung fibroblast cells. Confluent cultures of cells were serum starved for 24 h, before treatment with the indicated concentrations (50 and 100 μg/ml) of THSG for 24 h, and then stimulated with 2.5 ng/ml of TGF-β1 for 1 h (for TGFBR1 and TGFBR2), 6 h (for Smad2/3, Akt, ERK1/2, p38, and JNK), or 24 h (for mTOR). Whole cell lysates were prepared at the indicted times, and analyzed by Western blotting. The β-actin was used as a loading control. **(A)** Representative blots of TGFBR1 and TGFBR2 are shown. The relative protein levels of **(B)** TGFBR1 and **(C)** TGFBR2 were quantified by densitometry and normalized to β-actin. **(D)** Representative blots of p-Smad2/3, Smad2/3, p-Akt and Akt are shown. Quantification of **(E)** p-Smad2/3 and **(F)** p-Akt proteins was achieved by densitometry with reference to the respective Smad2/3 and Akt proteins. **(G)** Representative blots of p-mTOR and mTOR are shown. **(H)** Quantification of p-mTOR protein was achieved by densitometry and normalized to mTOR protein. **(I)** Representative blots of p-ERK1/2, ERK1/2, p-p38, p38, p-JNK, and JNK are shown. Quantification of **(J)** p-ERK1/2, **(K)** p-p38, and **(L)** p-JNK proteins was achieved by densitometry with reference to the respective ERK1/2, p38, and JNK proteins. Data are represented as the mean ± SEM of three independent experiments. ^#^ *p* < 0.05 compared with untreated group. *p < 0.05 compared with TGF-β1-treated group. (a) (b)

Since the TGF-β receptors (TGFBR1 and TGFBR2) represent important upstream regulators of the TGF-β/Smad signaling pathway, the effects of THSG on the expression of TGFBR1 and TGFBR2 proteins in TGF-β1-stimulated MRC-5 lung fibroblast cells were detected by Western blot analysis. The results showed that 2.5 ng/ml of TGF-β1 treatment for 1 h slightly upregulated the protein levels of TGFBR1 and TGFBR2. When cells were pretreated for 24 h with THSG (50 and 100 μg/ml) and then stimulated with TGF-β1 for 1 h, the protein level of TGFBR2 was obviously reduced while the protein level of TGFBR1 was not affected (Figures 3A–C). Similarly, pretreatment of THSG for 2 h markedly reduced TGF-β1-induced up-regulation of the expression of TGFBR2 in A549 lung alveolar epithelial cells, but did not decrease the protein level of TGFBR1 (Supplementary Figures S2A,B,C). TGF-β1-induced fibrotic effects though activation of the canonical Smad signaling pathway in most target cells is well documented. Thus, Western blot analysis was performed to further examine the effect of THSG against TGF-β1-induced activation of Smad signaling pathway. MRC-5 cells were pretreated with THSG (50 and 100 μg/ml) for 24 h and treated with 2.5 ng/ml of TGF-β1 for 1 h. The results demonstrated that TGF-β1 induced Smad2/3 phosphorylation, however, THSG pretreatment significantly dampened TGF-β1-induced Smad2/3 phosphorylation in a dose dependent manner (Figures 3D,E). Additionally, we observed that THSG dramatically inhibited TGF-β1-induced Smad2/3 phosphorylation in A549 cells, as shown in Supplementary Figures S2A,D.

### 2,3,5,4′-tetrahydroxystilbene-2-O-β-D-glucoside inhibits transforming growth factor-beta 1-induced Akt, mTOR, and ERK1/2 phosphorylation

It has been reported that the non-Smad signaling pathways, including PI3K/Akt/mTOR and MAPK signaling pathways, are involved in the formation of pulmonary fibrosis (Ruan et al., 2020; Li et al., 2021). Thus, we examined whether THSG can inhibit TGF-β1-induced phosphorylation of Akt, mTOR, ERK1/2, p38, and JNK in MRC-5 human lung fibroblast cells (Figures 3D,F–L). Western blot analysis of cell lysates showed that the expression levels of p-Akt, p-mTOR, p-ERK1/2, p-p38, and p-JNK were enhanced in TGF-β1-stimulated lung fibroblasts compared to the untreated cells. Nevertheless, THSG treatment significantly reduced the expression levels of p-Akt, p-mTOR, and p-ERK1/2, but did not diminish the expression levels of p-p38 and p-JNK in TGF-β1-stimulated lung fibroblasts. Likewise, Western blot analysis also demonstrated that TGF-β1-induced phosphorylation of Akt, mTOR, and ERK1/2 was drastically reduced by THSG treatment in A549 human lung epithelial cells, while THSG had no inhibitory effect on the expression of p-p38 and p-JNK in TGF-β1-stimulated lung epithelial cells (Supplementary Figures S2E–L).

### 2,3,5,4′-tetrahydroxystilbene-2-O-β-D-glucoside suppresses oxidative stress in MRC-5 human lung fibroblast cells

**FIGURE 4:**
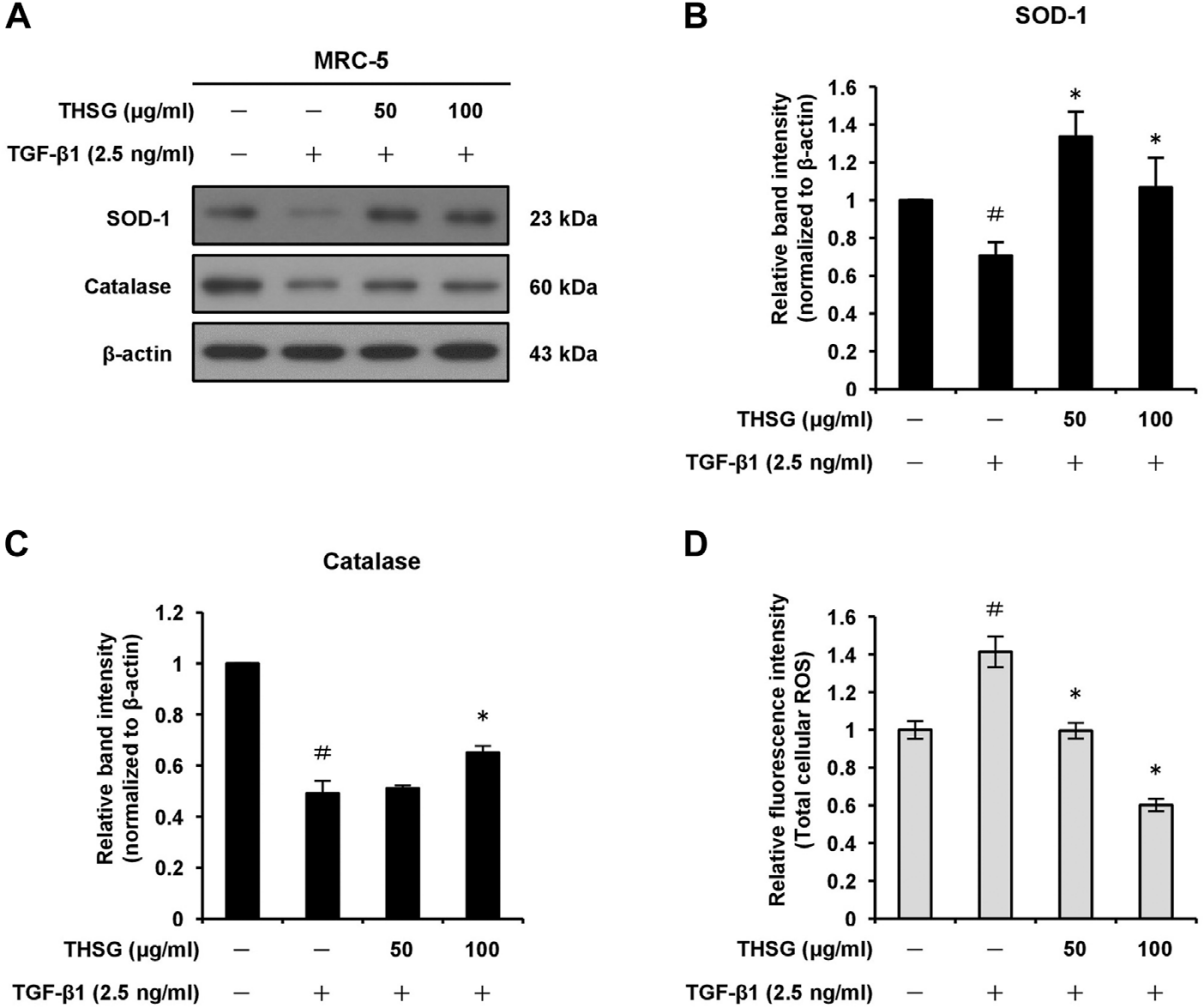
Effect of THSG on antioxidative enzymes expression and oxidative stress in TGF-β1-treated MRC-5 cells. **(A)** The cells were serum starved for 24 h prior to treatment with various concentrations (50 and 100 μg/ml) of THSG for 24 h, and then incubated with 2.5 ng/ml of TGF-β1 for a further 24 h. The protein expression levels of antioxidative enzymes SOD-1 and catalase in whole cell lysates by Western blotting. β-actin was used as a loading control. The relative protein levels of **(B)** SOD-1 and **(C)** catalase were quantified by densitometry and normalized to β-actin. **(D)** ROS production in cells subjected to different treatment was measured using a ROS detection kit. Data are represented as the mean ± SEM of three independent experiments. ^#^ *p* < 0.05 compared with untreated group. *p < 0.05 compared with TGF-β1-treated group.

In order to find out whether THSG is able to induce oxidative stress in MRC-5 cells, we examined the effect of THSG on the expression levels of anti-oxidant enzymes SOD-1 and catalase in TGF-β1-stimulated MRC-5 cells using Western blot analysis. The results showed that the treatment of 2.5 ng/ml TGF-β1 decreased the protein levels of SOD-1 and catalase in MRC-5 cells as compared to the untreated cells. However, the pretreatment of THSG (50 and 100 μg/ml) for 24 h efficiently increased the expression levels of SOD-1 and catalase proteins as compared to the TGF-β1-only treated cells (Figures 4A–C). The intracellular production of ROS in MRC-5 cells after the treatments were measured using the total ROS/Superoxide detection kit. After 24 h incubation of MRC-5 cells with 2.5 ng/ml TGF-β1, a significant increase in ROS production was observed when compared with the untreated group. In contrast, pretreatment of the cells with THSG in doses of 50 and 100 μg/ml markedly reduced the intracellular ROS generation in comparison to the TGF-β1-only treated group (Figure 4D).

### 2,3,5,4′-tetrahydroxystilbene-2-O-β-D-glucoside decreases transforming growth factor-beta 1 expression and increases SOD-1 and catalase expression in the lung tissues of mice with bleomycin-induced pulmonary fibrosis

**FIGURE 5:**
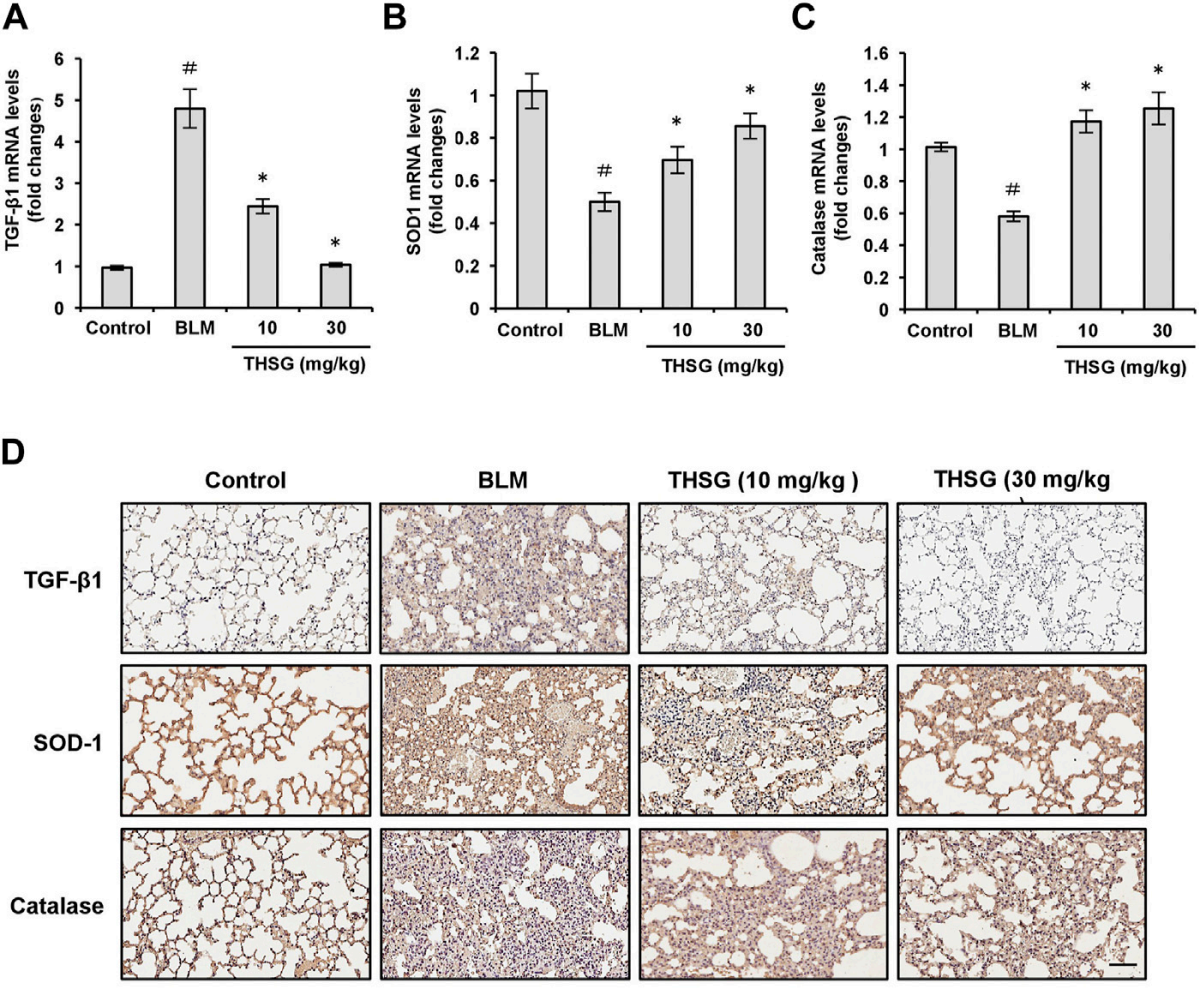
Effect of THSG on TGF-β1, SOD-1, and catalase expression in the lung tissues of BLM-stimulated mice. The mRNA levels of TGF-β1 **(A)**, SOD-1 **(B)**, and catalase **(C)** in the lung tissues of mice from each group on day 21 were detected by the qRT-PCR. **(D)** Representative photographs of immunohistochemical staining for TGF-β1, SOD-1, and catalase in the lung sections of mice from different experimental groups. Scale bar: 100 μm. Data are represented as the mean ± SEM (*n* = 6 in each group*).* ^#^ *p* < 0.05 compared with vehicle-treated control group. *p < 0.05 compared with BLM group.

TGF-β1 is a profibrotic mediator involved in myofibroblast differentiation and induction of ECM deposition. We assessed the expression level of TGF-β1 in the lung tissues of mice from all treatment groups by quantitative real-time polymerase chain reaction (qRT-PCR) and immunohistochemistry. Quantitative RT-PCR results showed that the mRNA level of TGF-β1 from lung tissues of mice after administration of BLM was significantly elevated compared with the untreated group, which was evidently suppressed by the treatment of THSG in a dose-dependent manner, as shown in Figure 5A. These results were confirmed by immunohistochemistry (Figure 5D). BLM administration produced a significant increase in TGF-β1 expression in lung tissues, but the increase was reduced by THSG treatment. Additionally, in order to validate the anti-oxidative effect of THSG, oxidative stress was evaluated by detecting the levels of SOD-1 and catalase in lung tissues. A significant inhibition of mRNA levels of SOD-1 and catalase was detected in the lung tissues of BLM-administrated mice as compared to the untreated group. THSG treatment restored BLM-induced reduction in the mRNA levels of SOD-1 and catalase (Figures 5B,C). Immunohistochemical staining also revealed that SOD-1 and catalase expression was reduced by BLM. In contrast, treatment with THSG increased the expression levels of SOD-1 and catalase (Figure 5D).

### 2,3,5,4′-tetrahydroxystilbene-2-O-β-D-glucoside induces autophagy activation in transforming growth factor-beta 1-stimulated MRC-5 human lung fibroblast cells

**FIGURE 6:**
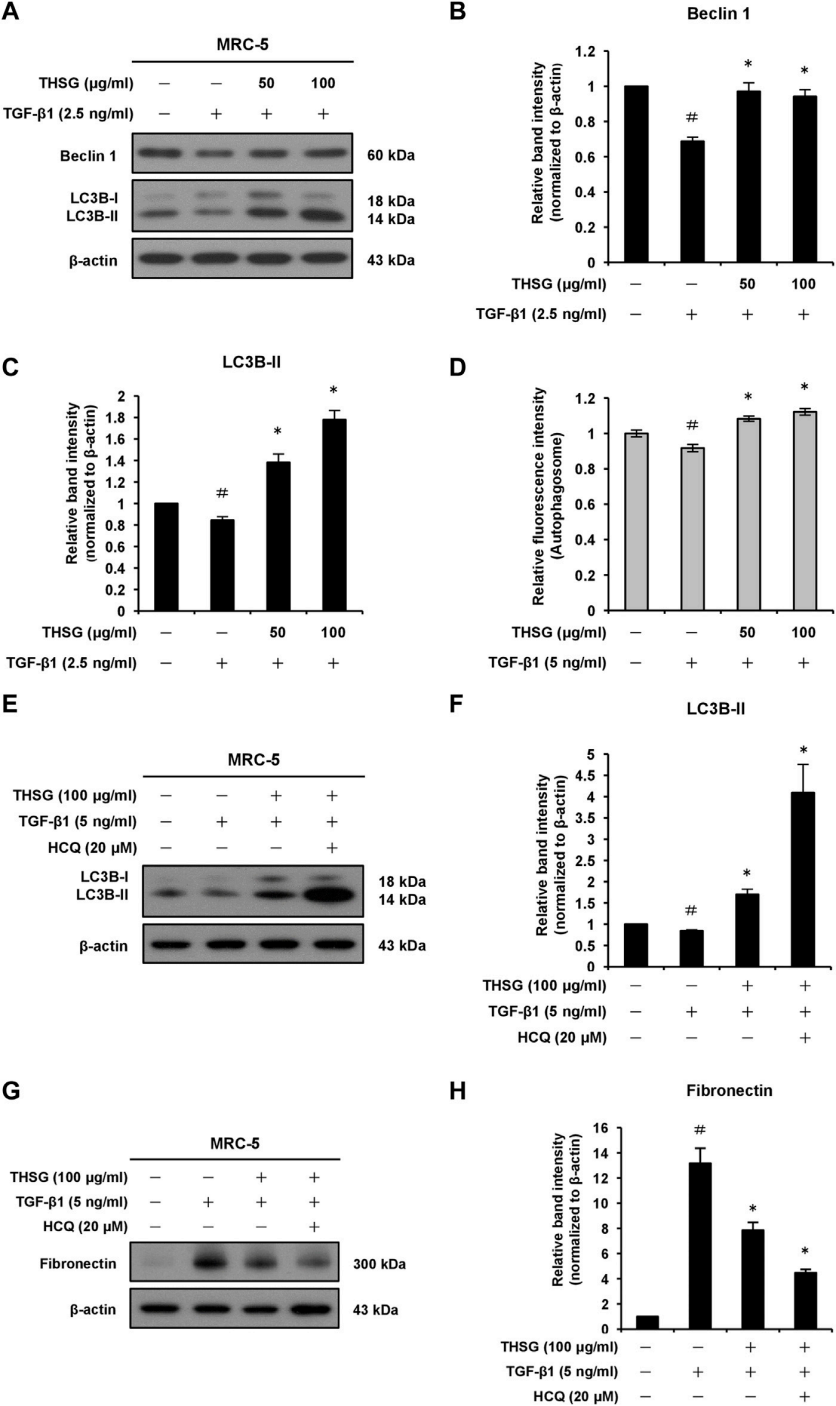
Effect of THSG on autophagy in MRC-5 cells treated with TGF-β1. **(A)** The cells were serum starved for 24 h, before treatment with various concentrations (50 and 100 μg/ml) of THSG for 24 h, and then incubated with 2.5 ng/ml of TGF-β1 for an additional 24 h. The protein expression levels of autophagic markers Beclin 1 and LC3B in whole cell lysates were assessed by Western blotting. The β-actin was used as a loading control. The relative protein expression levels of **(B)** Beclin 1 and **(C)** LC3B-II were quantified by densitometry and normalized to β-actin. **(D)** After treatment with the indicated concentrations of THSG for 24 h, and autophagy in cells was measured using an autophagy assay kit. **(E)** Cells were pretreated with THSG (100 μg/ml) for 24 h, and then subsequently stimulated with or without TGF-β1 (5 ng/ml) or HCQ (20 μM) for 24 h. The protein samples were collected after the treatment. Representative images of Western blotting showed the protein expression of LC3B. **(F)** The relative protein expression levels of LC3B-II were quantified by densitometry. **(G)** Representative images of Western blotting showed the protein expression of fibronectin. **(H)** Densitometric analysis showed the quantification of fibronectin expression. Data are represented as the mean ± SEM of three independent experiments. ^#^ *p* < 0.05 compared with untreated group. *p < 0.05 compared with TGF-β1-treated group.

To define whether THSG affects autophagy in MRC-5 human lung fibroblast cells. Autophagy was monitored by the expression levels of autophagy-associated proteins Beclin 1 and LC3B using Western blot analysis. As shown in Figures 6A–C, the expression levels of Beclin 1 and LC3B-II proteins were significantly decreased in the TGF-β1-treated group in comparison with the untreated group. However, the levels of Beclin 1 and LC3B-II proteins were significantly increased in the THSG-treated groups (cells were treated with THSG in doses of 50 and 100 μg/ml, followed by stimulation with 2.5 ng/ml of TGF-β1) compared with that in the TGF-β1-only treated group. In addition, the protein level of LC3B was measured after pretreated with 50 and 100 μg/ml of THSG for 2 h and then cotreated with 5 ng/ml of TGF-β1 for 24 h in A549 human lung alveolar epithelial cells. The protein expression level of LC3B-II was significantly increased in THSG-treated groups compared to the TGF-β1-only treated group (Supplementary Figures S3A,B). To confirm this notion, we used an autophagy assay kit. As indicated in Figure 6D, autophagosome formation decreased in TGF-β1-treated MRC-5 cells compared to the untreated cells, while pretreatment of these cells with 50 and 100 μg/ml of THSG significantly increased the fluorescent intensity of autophagosomes. In addition, studies have found that treatment of chloroquine (as an inhibitor of autophagy, which inhibits lysosome fusion with the autophagosome and thereby interferes autophagic degradation process) can enhance LC3-II expression and increase the autophagosome accumulation in TGF-β1-stimulated lung fibroblasts (Rangarajan et al., 2016; Liu et al., 2021). Therefore, we sought to demonstrate whether THSG restores TGF-β1-induced impaired autophagy, inhibition assay by using HCQ to examine the effect of THSG is related to autophagy inhibition or an increase in autophagic flux. After adding HCQ, the expression level of LC3B-II was increased in THSG-treated cells (Figures 6E,F). Moreover, the expression of LC3B-II was significantly higher than in THSG-treated cells, indicating the enhanced autophagosome formation and an increase in autophagic flux. To further test the effect of HCQ on the expression of fibrotic marker protein induced by TGF-β1 in THSG-treated lung fibroblasts. We found that HCQ augmented the downregulation of fibronectin by THSG (Figures 6G,H). Based on the above results, THSG-induced autophagy may partly account for the inhibitory effect of ECM proteins.

### 2,3,5,4′-tetrahydroxystilbene-2-O-β-D-glucoside treatment protects against pulmonary fibrosis induced by bleomycin

**FIGURE 7:**
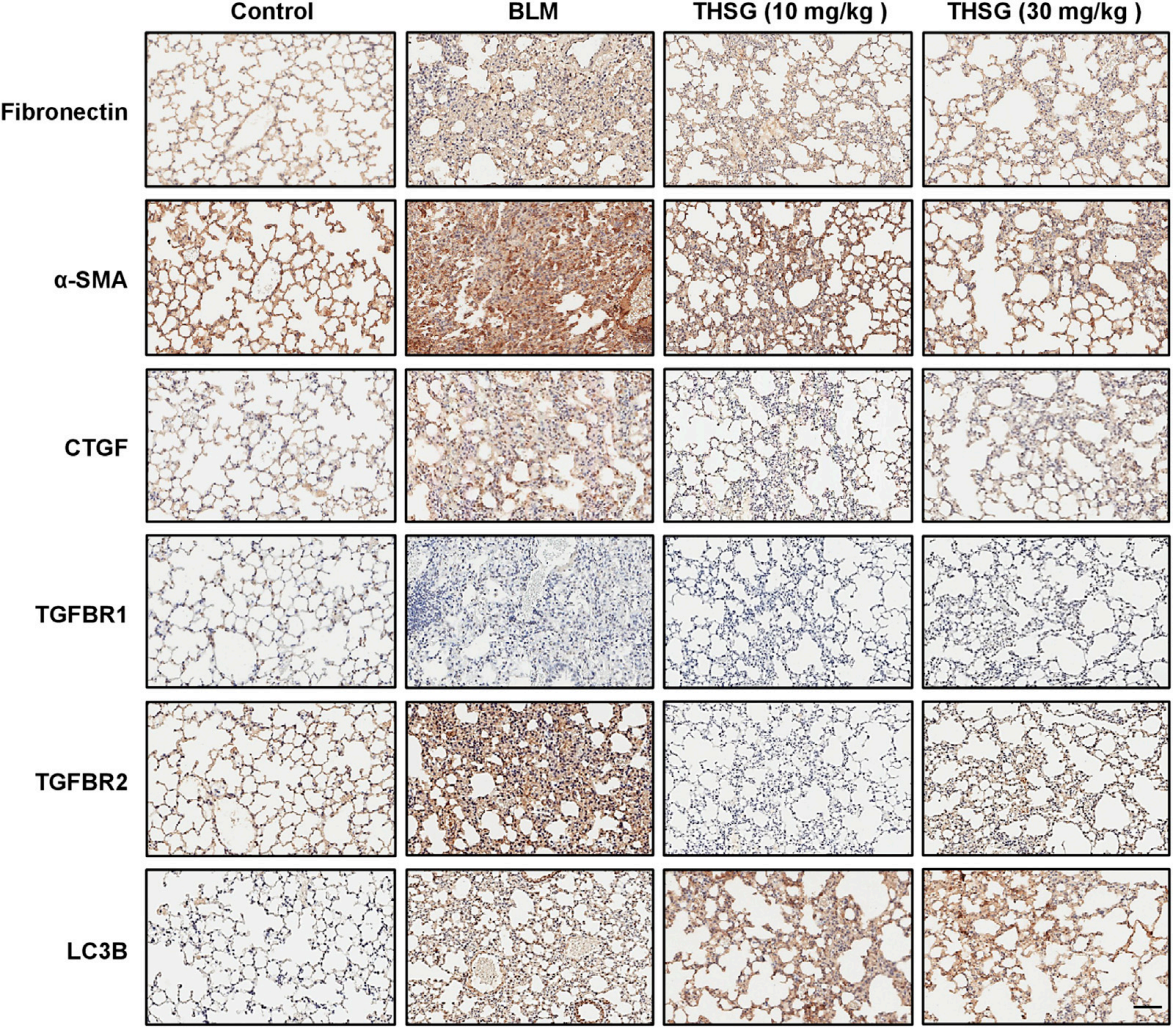
Effect of THSG on fibronectin, α-SMA, CTGF, TGFBR1, TGFBR2, and LC3B expression in the lung tissues of BLM-stimulated mice on day 21. Representative photographs of immunohistochemical staining for fibronectin, α-SMA, CTGF, TGFBR1, TGFBR2, and LC3B in the lung sections of mice from different experimental groups. Scale bar: 100 μm.

To assess the effect of THSG on the expression of fibronectin, α-SMA, CTGF, TGFBR1, TGFBR2, and LC3B in the lung tissue sections of mice from each group sacrificed on day 21, immunohistochemical staining was used to determine the protein expressions of these molecules. As shown in Figure 7, the protein levels of fibronectin, α-SMA, CTGF, and TGFBR2 were significantly increased in the lung tissues of BLM-treated mice, In contrast, treatment with THSG (10 or 30 mg/kg) exhibited a reduced levels of fibronectin, α-SMA, CTGF, and TGFBR2 in the lung tissues of mice compared to those of BLM-treated mice. However, the expression of LC3B was markedly increased in the lung tissues of THSG-treated mice compared to the BLM-treated mice. Additionally, there were no obvious changes in the expression of TGFBR1 in the lung tissues of mice from each group.

## Discussion

This study showed that THSG could alleviate lung fibrosis by suppressing ECM protein production and myofibroblast differentiation. Furthermore, THSG administration significantly ameliorates lung fibrosis in BLM-treated mice, which is similar to THSG therapeutic effects as depicted in another disease model such as fibrosis of heart and liver (Peng et al., 2016; Long et al., 2019). In a rat model of pressure overloaded-induced cardiac fibrosis model, THSG administration significantly attenuated the upregulated protein levels of pro-fibrotic markers such as fibronectin, type I and type III collagen induced by pressure overload (Peng et al., 2016). THSG treatment also markedly decreased the protein levels of fibronectin, type I and type III collagen induced by angiotensin II in rat cardiac fibroblasts (Peng et al., 2016). Likewise, in a rodent model of carbon tetrachloride (CCl4)-induced liver fibrosis, THSG significantly downregulated the expression of α-SMA protein in the liver tissues of rats (Long et al., 2019). In our research, treatment of THSG reduced the expression levels of α-SMA and fibronectin and collagen deposition in the lung tissues of mice exposed to BLM. THSG also decreased the protein levels of α-SMA and fibronectin in TGF-β1-stimulated MRC-5 lung fibroblast cells. Moreover, THSG treatment significantly inhibited TGF-β expression induced by CCl4 in rat liver tissue sections (Long et al., 2019). In a mice model of streptozotocin (STZ)-induced diabetes, THSG therapy dramatically suppressed the expression of fibronectin, CTGF, and TGF-β in kidney tissues and prevented renal injury and fibrosis (Chen et al., 2016). Similarly, our current results revealed that THSG administration effectively reduced the expression levels of TGF-β1 and CTGF induced by BLM in lung tissues of mice.

It has been reported that the protective effect of THSG against hepatic fibrosis was exerted by strikingly decreasing CCl4-induced phosphorylation of Smad2 and ERK1/2 in the liver tissues of rats (Long et al., 2019). Another study has shown that the phosphorylation of Smad3 was dramatically elevated in palmitic acid-stimulated cardiomyocytes, while THSG treatment significantly inhibited the up-regulation of p-Smad3 by palmitic acid (Zou and Kong, 2019). Moreover, increasing evidences have affirmed that the impediment of activation of Smad-dependent and -independent cascades including MAPK and PI3K/Akt/mTOR pathways, which effectively inhibited pulmonary fibrosis induced by BLM (Chitra et al., 2015; Liu et al., 2016; Qian et al., 2018). In harmony with these reports, the present study showed that the Smad2/3, Akt/mTOR, ERK1/2 signaling pathways were activated in TGF-β1-mediated fibrogenic response of human MRC-5 lung fibroblast cells and A549 lung alveolar epithelial cells, whereas THSG treatment inhibited TGF-β1-induced the phosphorylation of Smad2/3, ERK1/2, Akt, and mTOR. Accordingly, THSG efficiently suppressed TGF-β1-induced the expression of α-SMA, fibronectin, and CTGF in MRC-5 lung fibroblast cells and the expression of fibronectin and N-cadherin in A549 lung alveolar epithelial cells by blocking Smad2/3, ERK1/2, and Akt/mTOR signaling pathways. Overall, these results indicate potential advantages of THSG in the treatment of pulmonary fibrosis diseases.

Oxidative stress plays a crucial role in pulmonary fibrosis development and acts as a mediator of fibrogenic effect of TGF-β (Liu and Gaston Pravia, 2010). Several studies have shown that BLM administration triggers overproduction of ROS and an observed decrease in the levels of antioxidant (Huang et al., 2015; Zhao et al., 2020). Additionally, TGF-β1 stimulation causes an appreciable increase in ROS generation in MRC-5 human lung fibroblast cells (Fang et al., 2021). Therefore, we believe that anti-fibrogenic effect of THSG may be acted by repression of oxidative stress. In this study, we demonstrated that ROS production was substantially increased while the expression levels of SOD-1 and catalase were reduced in TGF-β1-stimulated MRC-5 human lung fibroblast cells. In contrast, THSG administration remarkably decreased the generation of ROS and efficiently enhanced the expression levels of SOD-1 and catalase. Moreover, our *in vivo* results showed that THSG increased the mRNA and protein levels of SOD-1 and catalase in lung tissues of BLM-treated mice. Similarly, in human skin fibroblasts, THSG treatment exhibited protective effects on UVB-induced premature senescence by increasing SOD level and suppressing oxidative stress (Xu and Wang, 2019). In another study, researchers reported that THSG attenuated gentamicin-induced ototoxicity in mouse cochlear UB/OC-2 cells by suppressing ROS generation and increasing the SOD activity (Wen et al., 2020). Wang et al. confirmed that THSG treatment significantly attenuated liver injury in prediabetic rats by increasing the activity of glutathione peroxidase (GPx) and SOD (Wang et al., 2020). These results indicate that THSG can depress lung fibrosis and injury induced by BLM through reducing oxidative stress.

THSG treatment has been proven to induce autophagy by upregulation of autophagy-related proteins Beclin 1, LC3-II, and ATG12 in the liver tissues of prediabetic rats (Wang et al., 2020). It was also reported that THSG significantly induced autophagy and increased the expression of LC3-II protein in human WRL-68 hepatic cells (Yang et al., 2020). Consistent with those reports above, our results revealed that THSG treatment dramatically increased the protein levels of Beclin 1 and LC3B-II in TGF-β1-stimulated MRC-5 human lung fibroblast cells. THSG treatment also increased LC3B-II protein expression in TGF-β1-stimulated A549 human lung alveolar epithelial cells. Besides, THSG administration increased the expression level of LC3B in the lung tissue sections of mice treated with BLM. Recent studies have shown that downregulation of p-mTOR and upregulation of LC3-II protein level, contributing to suppression of collagen deposition and EMT in primary lung fibroblasts and TGF-β1-induced lung epithelial cells, resulting in an alleviation of lung injury and improving lung function in BLM-induced animal models (Alsayed et al., 2022; Pei et al., 2022). Accumulating evidence has also shown that the repression of TGF-β1-induced activation of mTOR signaling in lung fibroblasts, which results in the induction of autophagy and the inhibition of myofibroblast activation and ECM production in mice model of BLM-induced pulmonary fibrosis (Li et al., 2021; Lu et al., 2022). Furthermore, THSG has been demonstrated to trigger autophagy in human hepatocytes via the inhibition of the PI3K/Akt/mTOR signaling pathway (Yang et al., 2020). Herein, these findings indicate that THSG inhibits the TGF-β1-induced Akt/mTOR pathway in human lung fibroblasts and alveolar epithelial cells, thereby leading to autophagy activation and subsequent alleviating lung fibrosis.

In conclusion, we demonstrate that THSG can effectively ameliorate BLM-induced pulmonary fibrosis in mice. We also provide *in vitro* evidence that THSG inhibits TGF-β1-induced myofibroblast differentiation, ECM production, and EMT-like process in human lung fibroblasts and alveolar epithelial cells. In particular, the protective effect of THSG against BLM-induced pulmonary fibrosis is not only relevant to its anti-oxidative and anti-fibrotic properties but also autophagy activation. The potential molecular mechanisms responsible for the anti-fibrotic effect of THSG is due to repression of both Smad and non-Smad signaling pathways. Therefore, THSG may be a potential agent for the treatment of pulmonary fibrosis diseases.

## Data Availability

The original contributions presented in the study are included in the article/Supplementary Material, further inquiries can be directed to the corresponding author.

## Supplementary material

SUPPLEMENTARY FIGURE S1 Effect of THSG on the protein expression levels of TGF-β1-induced EMT related markers in A549 human lung alveolar epithelial cells. **(A)** Confluent cultures of cells were starved in medium containing 0.1% FBS for 24 h, before treatment with the indicated concentrations (50 and 100 μg/ml) of THSG for 2 h, and then stimulated with 5 ng/ml of TGF-β1 for a further 48 h. The expressions of epithelial phenotype marker (E-cadherin) and mesenchymal phenotype markers (fibronectin and N-cadherin) in whole cell lysates were determined by Western blotting. β-actin was used as a loading control. Representative blots of fibronectin, N-cadherin, and E-cadherin are shown. The relative protein levels of **(B)** fibronectin, **(C)** N-cadherin, and **(D)** E-cadherin were quantified by densitometry and normalized to β-actin. The results were expressed as relative units. Data are represented as the mean ± SEM of three independent experiments. #*p* < 0.05 compared with untreated group. **p* < 0.05 compared with control (TGF-β1-treated) group.

SUPPLEMENTARY FIGURE S2 Effect of THSG on TGF-β receptors and TGF-β1-induced Smad-dependent and -independent signaling pathways in A549 human lung alveolar epithelial cells. The cells were starved for 24 h followed by treatment with the indicated doses (50 and 100 μg/ml) of THSG for 2 h, and then stimulated with 5 ng/ml of TGF-β1 for 30 min (for TGFBR1, TGFBR2, and Smad 2/3), 1 h (for Akt, ERK1/2, p38, and JNK), or 3 h (for mTOR). Whole cell lysates were prepared at the indicted times, and subjected to Western blotting. The β-actin was used as a loading control. **(A)** Representative blots of TGFBR1, TGFBR2, p-Smad2/3, and Smad2/3 are shown. The relative protein levels of **(B)** TGFBR1 and **(C)** TGFBR2 were quantified by densitometry and normalized to β-actin. **(D)** Quantification of p-Smad2/3 protein was achieved by densitometry and normalized to Smad2/3 protein. **(E)** Representative blots of p-Akt and Akt are shown. **(F)** Quantification of p-Akt protein was achieved by densitometry and normalized to Akt protein. **(G)** Representative blots of p-mTOR and mTOR are shown. **(H)** Quantification of p-mTOR was achieved by densitometry and normalized to mTOR. **(I)** Representative blots of p-ERK1/2, ERK1/2, p-p38, p38, p-JNK, and JNK are shown. Quantification of **(J)** p-ERK1/2, **(K)** p-p38, and **(L)** p-JNK proteins was analyzed by densitometry with reference to the respective ERK1/2, p38, and JNK proteins. Data are represented as the mean ± SEM of three independent experiments. #*p* < 0.05 compared with untreated group. **p* < 0.05 compared with TGF-β1-treated group.

SUPPLEMENTARY FIGURE S3 Effect of THSG on autophagy in A549 cells treated with TGF-β1. **(A)** The cells were starved for 24 h, before treatment with various concentrations (50 and 100 μg/ml) of THSG for 2 h, and then incubated with 5 ng/ml of TGF-β1 for a further 48 h. The expression of autophagic marker LC3B in whole cell lysates were determined by Western blotting. β-actin was used as a loading control. Representative blots are shown. The relative protein expression level of **(B)** LC3B-II was quantified by densitometry and normalized to β-actin. Data are represented as the mean ± SEM of three independent experiments. #*p* < 0.05 compared with untreated group. **p* < 0.05 compared with TGF-β1-treated group.

## References

[B1] Ahn J. Y. Kim M. H. Lim M. J. Park S. Lee S. L. Yun Y. S. (2011). The inhibitory effect of ginsan on TGF-β mediated fibrotic process. J. Cell. Physiol. 226 (5), 1241–1247. 10.1002/jcp.22452 20945375

[B2] Allen J. T. Spiteri M. A. (2002). Growth factors in idiopathic pulmonary fibrosis: Relative roles. Respir. Res. 3 (1), 13. 10.1186/rr162 11806848 PMC64811

[B3] Alsayed H. A. Mohammad H. M. F. Khalil C. M. El-Kherbetawy M. K. Elaidy S. M. (2022). Autophagy modulation by irbesartan mitigates the pulmonary fibrotic alterations in bleomycin challenged rats: Comparative study with rapamycin. Life Sci. 303, 120662. 10.1016/j.lfs.2022.120662 35636582

[B4] Araya J. Kojima J. Takasaka N. Ito S. Fujii S. Hara H. (2013). Insufficient autophagy in idiopathic pulmonary fibrosis. Am. J. Physiol. Lung Cell. Mol. Physiol. 304 (1), L56–L69. 10.1152/ajplung.00213.2012 23087019

[B5] Ashcroft T. Simpson J. M. Timbrell V. (1988). Simple method of estimating severity of pulmonary fibrosis on a numerical scale. J. Clin. Pathol. 41 (4), 467–470. 10.1136/jcp.41.4.467 3366935 PMC1141479

[B6] Bao Z. Zhang Q. Wan H. He P. Zhou X. Zhou M. (2014). Expression of suppressor of cytokine signaling 1 in the peripheral blood of patients with idiopathic pulmonary fibrosis. Chin. Med. J. 127 (11), 2117–2120. 24890164

[B7] Bouros D. Antoniou K. M. (2005). Current and future therapeutic approaches in idiopathic pulmonary fibrosis. Eur. Respir. J. 26 (4), 693–702. 10.1183/09031936.05.00145004 16204603

[B8] Büchter C. Zhao L. Havermann S. Honnen S. Fritz G. Proksch P. (2015). TSG (2, 3, 5, 4'-Tetrahydroxystilbene-2-O-β-D-glucoside) from the Chinese herb *Polygonum multiflorum* increases life span and stress resistance of *Caenorhabditis elegans* . Oxid. Med. Cell. Longev., 124357. 10.1155/2015/124357 26075030 PMC4436517

[B9] Cabrera S. Maciel M. Herrera I. Nava T. Vergara F. Gaxiola M. (2015). Essential role for the ATG4B protease and autophagy in bleomycin-induced pulmonary fibrosis. Autophagy 11 (4), 670–684. 10.1080/15548627.2015.1034409 25906080 PMC4502665

[B10] Chen G. T. Yang M. Chen B. B. Song Y. Zhang W. Zhang Y. (2016). 2, 3, 5, 4'-Tetrahydroxystilbene-2-O-β-D-glucoside exerted protective effects on diabetic nephropathy in mice with hyperglycemia induced by streptozotocin. Food Funct. 7 (11), 4628–4636. 10.1039/c6fo01319h 27747335

[B11] Chitra P. Saiprasad G. Manikandan R. Sudhandiran G. (2015). Berberine inhibits Smad and non-Smad signaling cascades and enhances autophagy against pulmonary fibrosis. J. Mol. Med. 93 (9), 1015–1031. 10.1007/s00109-015-1283-1 25877860

[B12] Fang L. Wang W. Chen J. Zuo A. Gao H. Yan T. (2021). Osthole attenuates bleomycin-induced pulmonary fibrosis by modulating NADPH oxidase 4-derived oxidative stress in mice. Oxid. Med. Cell.. Longev. 2021, 3309944. 10.1155/2021/3309944 34527170 PMC8437590

[B13] Fernandez I. E. Eickelberg O. (2012). The impact of TGF-β on lung fibrosis: From targeting to biomarkers. Proc. Am. Thorac. Soc. 9 (3), 111–116. 10.1513/pats.201203-023AW 22802283

[B14] Gibson C. D. Kugler M. C. Deshwal H. Munger J. S. Condos R. (2020). Advances in targeted therapy for progressive fibrosing interstitial lung disease. Lung 198 (4), 597–608. 10.1007/s00408-020-00370-1 32591895

[B15] Haspel J. A. Choi A. M. (2011). Autophagy: A core cellular process with emerging links to pulmonary disease. Am. J. Respir. Crit. Care Med. 184 (11), 1237–1246. 10.1164/rccm.201106-0966CI 21836133 PMC3262043

[B16] Huang T. T. Chong K. Y. Ojcius D. M. Wu Y. H. Ko Y. F. Wu C. Y. (2013). *Hirsutella sinensis* mycelium suppresses interleukin-1β and interleukin-18 secretion by inhibiting both canonical and non-canonical inflammasomes. Sci. Rep. 3, 1374. 10.1038/srep01374 23459183 PMC3587886

[B17] Huang T. T. Lai H. C. Ko Y. F. Ojcius D. M. Lan Y. W. Martel J. (2015). *Hirsutella sinensis* mycelium attenuates bleomycin-induced pulmonary inflammation and fibrosis *in vivo* . Sci. Rep. 5, 15282. 10.1038/srep15282 26497260 PMC4620496

[B18] Huang T. T. Wu S. P. Chong K. Y. Ojcius D. M. Ko Y. F. Wu Y. H. (2014). The medicinal fungus *Antrodia cinnamomea* suppresses inflammation by inhibiting the NLRP3 inflammasome. J. Ethnopharmacol. 155 (1), 154–164. 10.1016/j.jep.2014.04.053 24858059

[B19] Jarman E. R. Khambata V. S. Cope C. Jones P. Roger J. Ye L. Y. (2014). An inhibitor of NADPH oxidase-4 attenuates established pulmonary fibrosis in a rodent disease model. Am. J. Respir. Cell. Mol. Biol. 50 (1), 158–169. 10.1165/rcmb.2013-0174OC 23977848

[B20] Jung C. H. Ro S. H. Cao J. Otto N. M. Kim D. H. (2010). mTOR regulation of autophagy. FEBS Lett. 584 (7), 1287–1295. 10.1016/j.febslet.2010.01.017 20083114 PMC2846630

[B21] Koli K. Myllärniemi M. Keski-Oja J. Kinnula V. L. (2008). Transforming growth factor-β activation in the lung: Focus on fibrosis and reactive oxygen species. Antioxid. Redox Signal. 10 (2), 333–342. 10.1089/ars.2007.1914 17961070

[B22] Kothapalli D. Frazier K. S. Welply A. Segarini P. R. Grotendorst G. R. (1997). Transforming growth factor beta induces anchorage-independent growth of NRK fibroblasts via a connective tissue growth factor-dependent signaling pathway. Cell. Growth Differ. 8 (1), 61–68. 8993835

[B23] Li M. Krishnaveni M. S. Li C. Zhou B. Xing Y. Banfalvi A. (2011). Epithelium-specific deletion of TGF-β receptor type II protects mice from bleomycin-induced pulmonary fibrosis. J. Clin. Investig. 121 (1), 277–287. 10.1172/JCI42090 21135509 PMC3007138

[B24] Li X. Ma L. Huang K. Wei Y. Long S. Liu Q. (2021). Regorafenib-attenuated, bleomycin-induced pulmonary fibrosis by inhibiting the TGF-β1 signaling pathway. Int. J. Mol. Sci. 22 (4), 1985. 10.3390/ijms22041985 33671452 PMC7922359

[B25] Ling S. Xu J. W. (2016). Biological activities of 2, 3, 5, 4’-tetrahydroxystilbene-2 O-β-D-glucoside in antiaging and antiaging related disease treatments. Oxid. Med. Cell. Longev., 4973239. 10.1155/2016/4973239 27413420 PMC4931083

[B26] Liu Q. Chu H. Ma Y. Wu T. Qian F. Ren X. (2016). Salvianolic acid B attenuates experimental pulmonary fibrosis through inhibition of the TGF-β signaling pathway. Sci. Rep. 6, 27610. 10.1038/srep27610 27278104 PMC4899783

[B27] Liu R. M. Gaston Pravia K. A. (2010). Oxidative stress and glutathione in TGF-β-mediated fibrogenesis. Free Radic. Biol. Med. 48 (1), 1–15. 10.1016/j.freeradbiomed.2009.09.026 19800967 PMC2818240

[B28] Liu Y. Zhong W. Zhang J. Chen W. Lu Y. Qiao Y. (2021). Tetrandrine modulates rheb-mTOR signaling-mediated selective autophagy and protects pulmonary fibrosis. Front. Pharmacol. 12, 739220. 10.3389/fphar.2021.739220 34880752 PMC8645995

[B29] Long T. Wang L. Yang Y. Yuan L. Zhao H. Chang C. C. (2019). Protective effects of trans-2, 3, 5, 4'-tetrahydroxystilbene 2-O-β-D-glucopyranoside on liver fibrosis and renal injury induced by CCl _4_ via down-regulating p-ERK1/2 and p-Smad1/2. Food Funct. 10 (8), 5115–5123. 10.1039/c9fo01010f 31364649

[B30] Lu Y. Zhong W. Liu Y. Chen W. Zhang J. Zeng Z. (2022). Anti-PD-L1 antibody alleviates pulmonary fibrosis by inducing autophagy via inhibition of the PI3K/Akt/mTOR pathway. Int. Immunopharmacol. 104, 108504. 10.1016/j.intimp.2021.108504 35026657

[B31] Luo F. Zhuang Y. Sides M. D. Sanchez C. G. Shan B. White E. S. (2014). Arsenic trioxide inhibits transforming growth factor-β1-induced fibroblast to myofibroblast differentiation *in vitro* and bleomycin induced lung fibrosis *in vivo* . Respir. Res. 15 (1), 51. 10.1186/1465-9921-15-51 24762191 PMC4113202

[B32] Patel A. S. Lin L. Geyer A. Haspel J. A. An C. H. Cao J. (2012). Autophagy in idiopathic pulmonary fibrosis. PLoS One 7 (7), e41394. 10.1371/journal.pone.0041394 22815997 PMC3399849

[B33] Pei X. Zheng F. Li Y. Lin Z. Han X. Feng Y. (2022). Niclosamide ethanolamine salt alleviates idiopathic pulmonary fibrosis by modulating the PI3K-mTORC1 pathway. Cells 11 (3), 346. 10.3390/cells11030346 35159160 PMC8834116

[B34] Peng Y. Zeng Y. Xu J. Huang X. L. Zhang W. Xu X. L. (2016). PPAR-γ is involved in the protective effect of 2, 3, 4', 5-tetrahydroxystilbene-2-O-beta-D-glucoside against cardiac fibrosis in pressure-overloaded rats. Eur. J. Pharmacol. 791, 105–114. 10.1016/j.ejphar.2016.08.025 27568841

[B35] Qian W. Cai X. Qian Q. Zhang W. Wang D. (2018). Astragaloside IV modulates TGF-β1-dependent epithelial-mesenchymal transition in bleomycin-induced pulmonary fibrosis. J. Cell. Mol. Med. 22 (9), 4354–4365. 10.1111/jcmm.13725 29971947 PMC6111865

[B36] Raghu G. Rochwerg B. Zhang Y. Garcia C. A. Azuma A. Behr J. (2015). An official ATS/ERS/JRS/ALAT clinical practice guideline: Treatment of idiopathic pulmonary fibrosis. An update of the 2011 clinical practice guideline. Am. J. Respir. Crit. Care Med. 192 (2), e3–e19. 10.1164/rccm.201506-1063ST 26177183

[B37] Rangarajan S. Kurundkar A. Kurundkar D. Bernard K. Sanders Y. Y. Ding Q. (2016). Novel mechanisms for the antifibrotic action of nintedanib. Am. J. Respir. Cell. Mol. Biol. 54 (1), 51–59. 10.1165/rcmb.2014-0445OC 26072676 PMC4742925

[B38] Richeldi L. Collard H. R. Jones M. G. (2017). Idiopathic pulmonary fibrosis. Lancet 389 (10082), 1941–1952. 10.1016/S0140-6736(17)30866-8 28365056

[B39] Ruan H. Lv Z. Liu S. Zhang L. Huang K. Gao S. (2020). Anlotinib attenuated bleomycin-induced pulmonary fibrosis via the TGF-β1 signalling pathway. J. Pharm. Pharmacol. 72 (1), 44–55. 10.1111/jphp.13183 31659758

[B40] Saito M. Mitani A. Ishimori T. Miyashita N. Isago H. Mikami Y. (2020). Active mTOR in lung epithelium promotes epithelial–mesenchymal transition and enhances lung fibrosis. Am. J. Respir. Cell. Mol. Biol. 62 (6), 699–708. 10.1165/rcmb.2019-0255OC 32208980

[B41] Sauleda J. Núñez B. Sala E. Soriano J, B. (2018). Idiopathic pulmonary fibrosis: Epidemiology, natural history, phenotypes. Med. Sci. 6 (4), 110. 10.3390/medsci6040110 PMC6313500 30501130

[B42] Shi Y. Massagué J. (2003). Mechanisms of TGF-β signaling from cell membrane to the nucleus. Cell. 113 (6), 685–700. 10.1016/s0092-8674(03)00432-x 12809600

[B43] Sime P. J. Xing Z. Graham F. L. Csaky K. G. Gauldie J. (1997). Adenovector-mediated gene transfer of active transforming growth factor-beta1 induces prolonged severe fibrosis in rat lung. J. Clin. Investig. 100 (4), 768–776. 10.1172/JCI119590 9259574 PMC508247

[B44] Tanaka K. Ishihara T. Azuma A. Kudoh S. Ebina M. Nukiwa T. (2010). Therapeutic effect of lecithinized superoxide dismutase on bleomycin-induced pulmonary fibrosis. Am. J. Physiol. Lung Cell. Mol. Physiol. 298 (3), L348–L360. 10.1152/ajplung.00289.2009 20034962

[B45] Wang C. Dai S. Gong L. Fu K. Ma C. Liu Y. (2022). A review of pharmacology, toxicity and pharmacokinetics of 2, 3, 5, 4'-tetrahydroxystilbene-2-O-β-D-glucoside. Front. Pharmacol. 12, 791214. 10.3389/fphar.2021.791214 35069206 PMC8769241

[B46] Wang K. Zhang T. Lei Y. Li X. Jiang J. Lan J. (2018). Identification of ANXA2 (annexin A2) as a specific bleomycin target to induce pulmonary fibrosis by impeding TFEB-mediated autophagic flux. Autophagy 14 (2), 269–282. 10.1080/15548627.2017.1409405 29172997 PMC5902212

[B47] Wang X. Zeng J. Wang X. Li J. Chen J. Wang N. (2020). 2, 3, 5, 4'-tetrahydroxystilbene-2-O-β-D-glucoside induces autophagy of liver by activating PI3K/Akt and Erk pathway in prediabetic rats. BMC Complement. Med. Ther. 20 (1), 177. 10.1186/s12906-020-02949-w 32513151 PMC7278085

[B48] Wen Y. H. Lin J. N. Wu R. S. Yu S. H. Hsu C. J. Tseng G. F. (2020). Otoprotective effect of 2, 3, 4', 5-Tetrahydroxystilbene-2-O-β-d-Glucoside on gentamicin-induced apoptosis in mouse cochlear UB/OC-2 cells. Molecules 25 (13), 3070. 10.3390/molecules25133070 PMC7412181 32640539

[B49] Wilkes M. C. Mitchell H. Penheiter S. G. Doré J. J. Suzuki K. Edens M. (2005). Transforming growth factor-β activation of phosphatidylinositol 3-kinase is independent of Smad2 and Smad3 and regulates fibroblast responses via p21-activated kinase-2. Cancer Res. 65 (22), 10431–10440. 10.1158/0008-5472.CAN-05-1522 16288034

[B50] Wu T. Y. Lin J. N. Luo Z. Y. Hsu C. J. Wang J. S. Wu H. P. (2020). 2, 3, 4', 5-Tetrahydroxystilbene-2-O-β-D-glucoside (THSG) Activates the Nrf2 antioxidant pathway and attenuates oxidative stress-induced cell death in mouse cochlear UB/OC-2 cells. Biomolecules 10 (3), 465. 10.3390/biom10030465 PMC7175305 32197448

[B51] Xu J. Lamouille S. Derynck R. (2009). TGF-β-induced epithelial to mesenchymal transition. Cell. Res. 19 (2), 156–172. 10.1038/cr.2009.5 19153598 PMC4720263

[B52] Xu M. Wang Y. (2019). Stilbene glucoside inhibits ultraviolet radiation B-induced photoaging in human skin fibroblasts. J. Zhejiang Univ. Med. Sci. 48 (6), 625–630. 10.3785/j.issn.1008-9292.2019.12.06 PMC10412946 31955536

[B53] Yang L. Xing W. Xiao W. Z. Tang L. Wang L. Liu M. J. (2020). 2, 3, 5, 4'-Tetrahydroxy-stilbene-2-O-beta-D-glucoside induces autophagy-mediated apoptosis in hepatocytes by upregulating miR-122 and inhibiting the PI3K/Akt/mTOR pathway: Implications for its hepatotoxicity. Pharm. Biol. 58 (1), 806–814. 10.1080/13880209.2020.1803367 32881597 PMC8641687

[B54] Yu W. Zhang X. Wu H. Zhou Q. Wang Z. Liu R. (2017). HO-1 is essential for tetrahydroxystilbene glucoside mediated mitochondrial biogenesis and anti-inflammation process in LPS-treated RAW264.7 macrophages. Oxid. Med. Cell. Longev., 1818575. 10.1155/2017/1818575 28473878 PMC5394384

[B55] Zhang Y. E. (2009). Non-smad pathways in TGF-β signaling. Cell. Res. 19 (1), 128–139. 10.1038/cr.2008.328 19114990 PMC2635127

[B56] Zhao H. Li C. Li L. Liu J. Gao Y. Mu K. (2020). Baicalin alleviates bleomycin-induced pulmonary fibrosis and fibroblast proliferation in rats via the PI3K/AKT signaling pathway. Mol. Med. Rep. 21 (6), 2321–2334. 10.3892/mmr.2020.11046 32323806 PMC7185294

[B57] Zou Y. Kong M. (2019). Tetrahydroxy stilbene glucoside alleviates palmitic acid-induced inflammation and apoptosis in cardiomyocytes by regulating miR-129-3p/Smad3 signaling. Cell. Mol. Biol. Lett. 24, 5. 10.1186/s11658-018-0125-x 30820195 PMC6379973

