# Supplementary figures and images for: 2,3,5,4′-tetrahydroxystilbene-2-O-β-D-glucoside ameliorates bleomycin-induced pulmonary fibrosis via regulating pro-fibrotic signaling pathways
Source: Front Pharmacol. 2022 Oct 4;13:997100. doi: 10.3389/fphar.2022.997100 (PMC9577370; doi:10.3389/fphar.2022.997100)

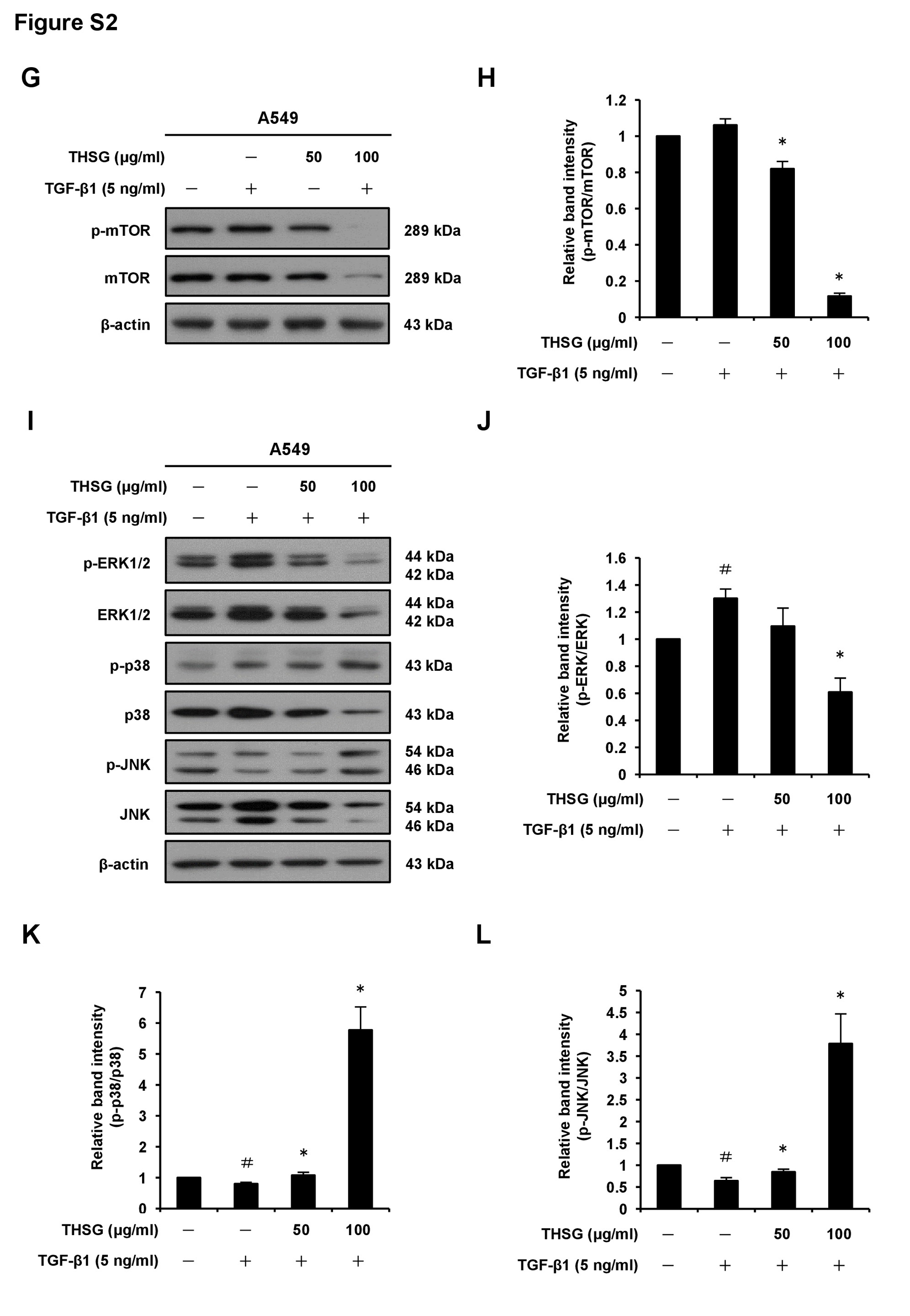

Supplement: Supplementary file 1 [file Image3.jpg]

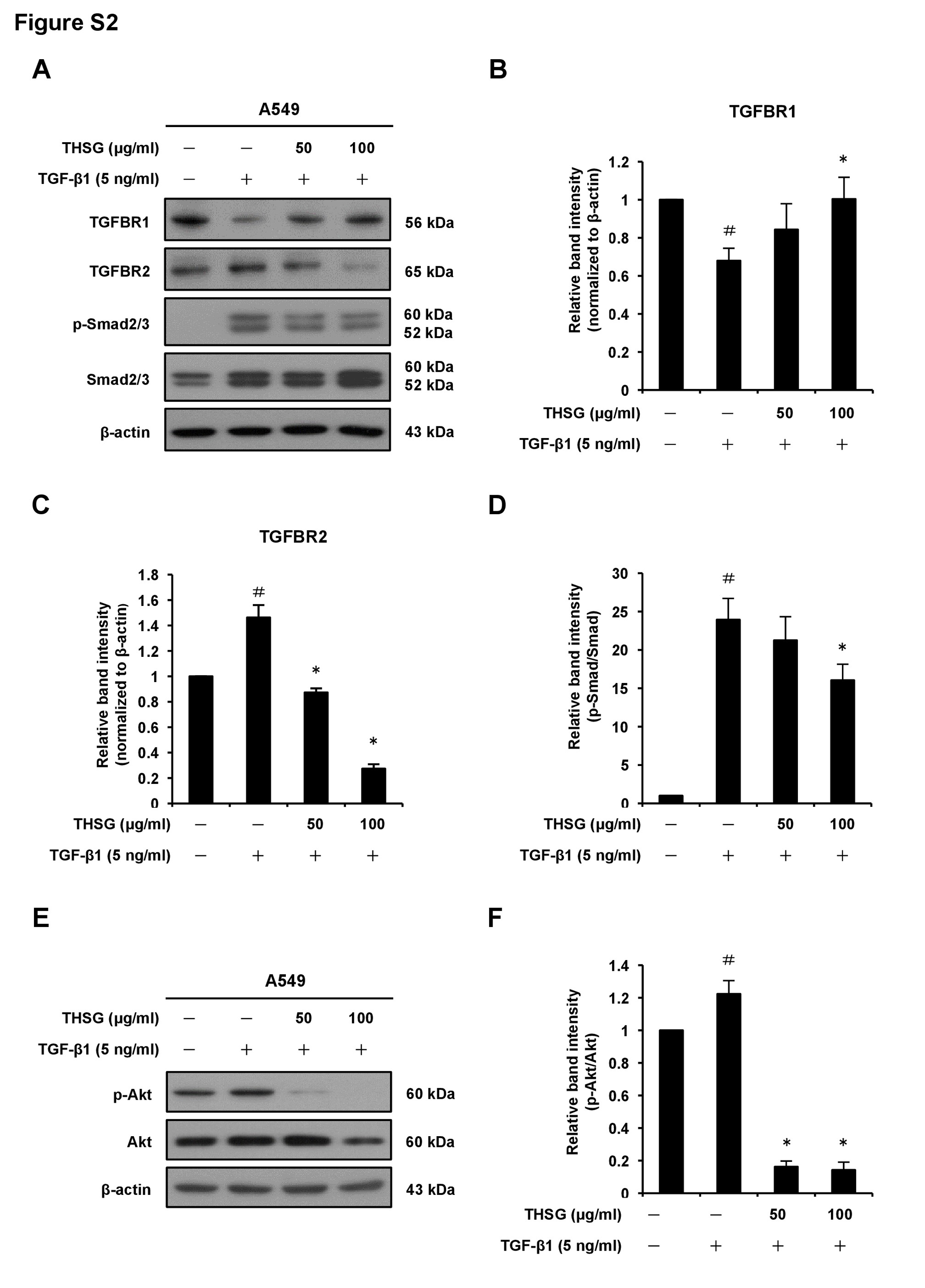

Supplement: Supplementary file 2 [file Image2.jpg]

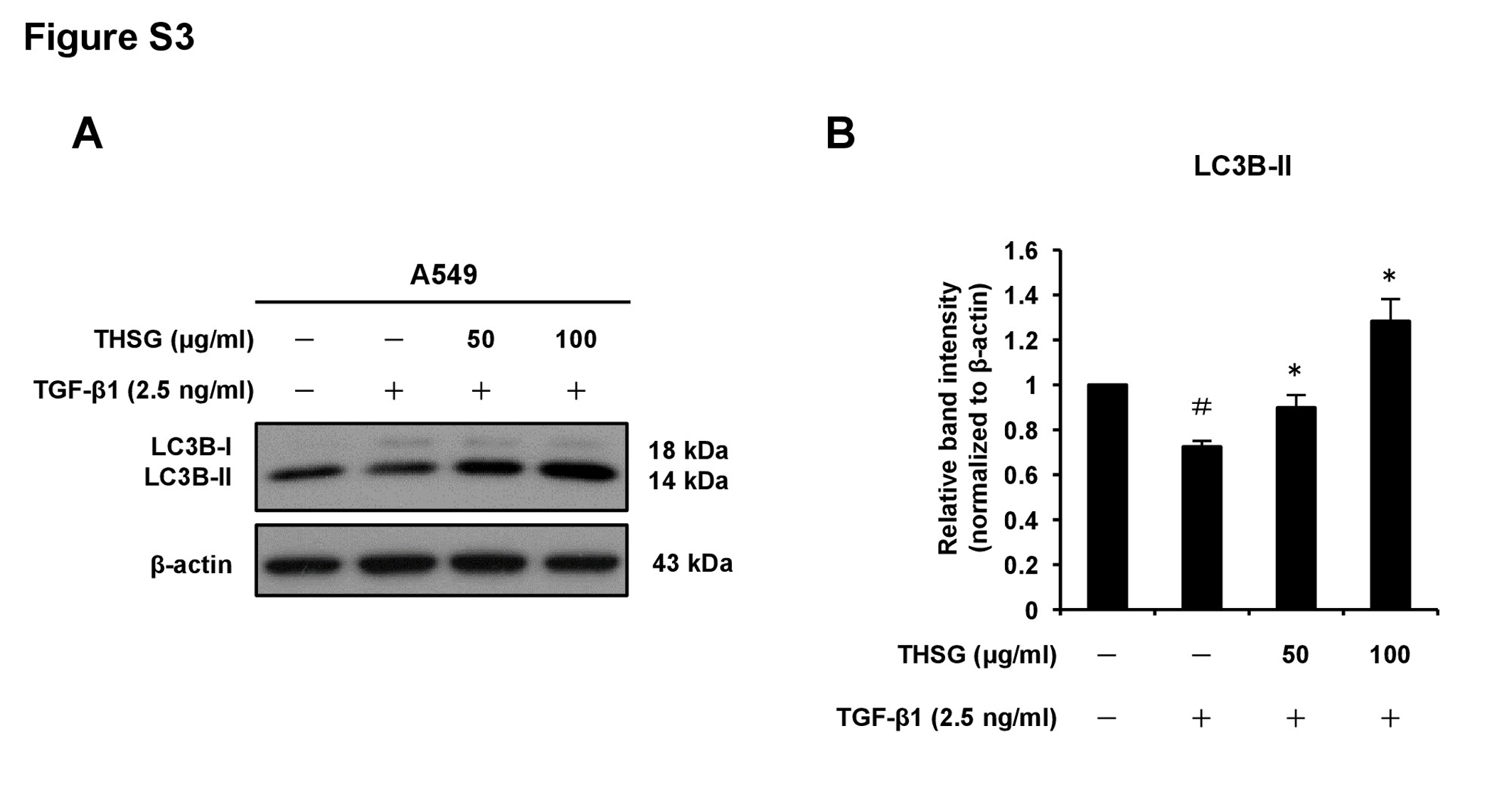

Supplement: Supplementary file 3 [file Image4.jpg]

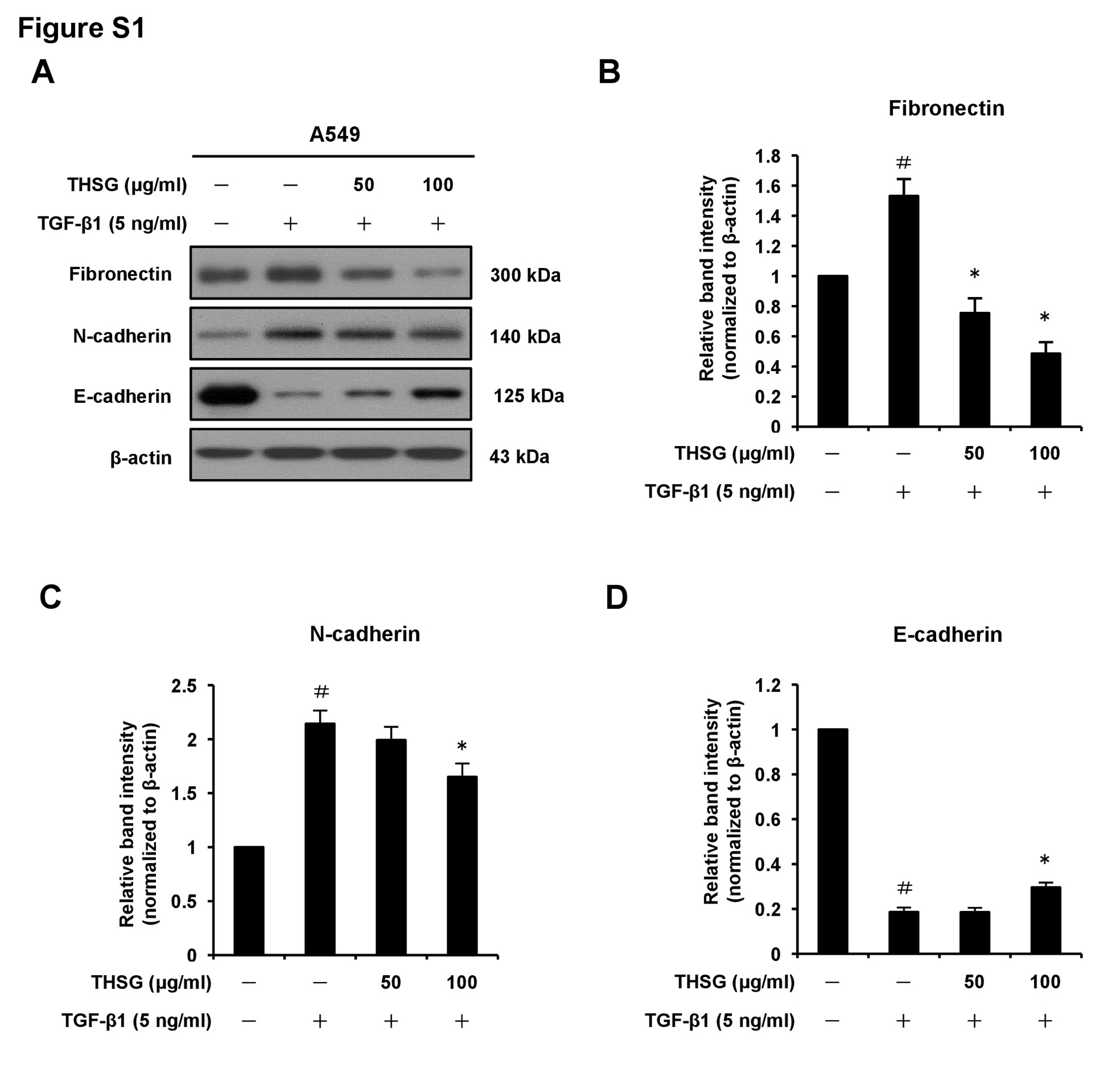

Supplement: Supplementary file 4 [file Image1.jpg]
